# Integrated Bioinformatics Analysis and Cellular Experimental Validation Identify Lipoprotein Lipase Gene as a Novel Biomarker for Tumorigenesis and Prognosis in Lung Adenocarcinoma

**DOI:** 10.3390/biology14050566

**Published:** 2025-05-19

**Authors:** Wanwan He, Meilian Wei, Yan Huang, Junsen Qin, Meng Liu, Na Liu, Yanli He, Chuanbing Chen, Yali Huang, Heng Yin, Ren Zhang

**Affiliations:** 1 Research Centre of Basic Integrative Medicine, School of Basic Medical Sciences, Guangzhou University of Chinese Medicine, Guangzhou 510006, China; hww199808@outlook.com (W.H.); weimeilian@stu.gzucm.edu.cn (M.W.); huangyan200310@163.com (Y.H.); 20241110004@stu.gzucm.edu.cn (J.Q.); liumeng@stu.gzucm.edu.cn (M.L.); liuna@gzucm.edu.cn (N.L.); blhhh@gzucm.edu.cn (Y.H.); huangyali@gzucm.edu.cn (Y.H.); 2 School of Pharmaceutical Sciences, Guangzhou University of Chinese Medicine, Guangzhou 510006, China; henchuanbing@gzucm.edu.cn; 3 Institute of Infectious Diseases, Guangzhou Medical University, Guangzhou 510182, China

**Keywords:** lipoprotein lipase, lung adenocarcinoma, SMR, targeted therapeutic, IncuCyte

## Abstract

Lung adenocarcinoma (LUAD) is one of the leading causes of death worldwide, and thus, more biomarker and therapeutic targets need to be explored. Herein, we aimed to explore new biomarkers of LUAD by integrating bioinformatics analysis with cell experiments. We firstly identified 266 druggable genes that were significantly differentially expressed between LUAD tissues and adjacent normal lung tissues. Among these genes, SMR analysis with *p*-value correction suggested that declining lipoprotein lipase (LPL) levels may be causally associated with an elevated risk of LUAD, which was corroborated by co-localization analysis. Analyses of clinical data showed that LPL in lung cancer tissues has considerable diagnostic value for LUAD, and elevated LPL levels were positively associated with improved patient survival outcomes. Cell experiments with an LPL activator proved these findings; the activator inhibited the proliferation and migration of lung cancer cells. Next, we found that LPL promoted the infiltration of immune cells such as DCs, IDCs, and macrophages in LUAD by mononuclear sequencing analysis and TIMER2.0. Meanwhile, patients with low levels of LPL expression demonstrated superior immunotherapeutic responses to anti-PD-1 therapy. We conclude that LPL acts as a diagnostic and prognostic marker for LUAD.

## Simple Summary

Lipoprotein lipase (LPL) is a key enzyme in lipid metabolism that hydrolyzes triglycerides, but its role in lung adenocarcinoma (LUAD) has not received much attention or research. In this study, we found that the LPL gene in lung tissue is negatively correlated with the risk of LUAD. We explored the role of LPL in LUAD through bioinformatics analysis and functional cell experiments. The results showed that it has a strong correlation with the infiltration of myeloid cells in the tumor microenvironment. Moreover, individuals diagnosed with LUAD and having lower LPL expression showed higher sensitivity to anti-PD-1 immune checkpoint inhibition therapy. Finally, activating LPL in cell experiments could inhibit the activity of LUAD cells. Therefore, we believe that LPL can serve as a novel biomarker for LUAD, and targeting this molecule for treatment may provide a new approach for LUAD therapy.

## 1. Introduction

Lung cancer continues to be a leading cause of cancer-related mortality worldwide [1], claiming millions of lives annually and representing a critical challenge to public health. Lung adenocarcinoma (LUAD) represents the most commonly diagnosed histological form of lung cancer in both male and female patients [2]. Currently, the incidence of LUAD among all lung cancer cases is on the rise, underscoring the growing need for the identification of effective therapeutic approaches. Despite remarkable progress in precision medicine for LUAD, which has significantly extended life expectancy in select LUAD populations through molecular-targeted approaches against driver mutations, including epidermal growth factor receptor (EGFR) and anaplastic lymphoma kinase (ALK) alterations, the majority of LUAD cases still remain without definitive, actionable targets [3]. Furthermore, the clinical application of targeted therapies faces significant challenges, especially given the limited number of drug targets available; currently, known therapeutic targets cover only a fraction of all LUAD cases, making it difficult to benefit every patient [4]. Therefore, identifying new genes for targeted therapy is crucial for expanding treatment coverage among this patient population.

The rapid advancement of genomics has opened new paths for the identification of drug targets for LUAD [5]. For example, bioinformatics approaches can be utilized to explore the inherent differences in gene expression within LUAD based on RNA-seq data, while Mendelian randomization utilizes genetic variants as instrumental factors to deduce potential causal associations. This methodology overcomes the inherent limitations of observational studies, thereby enhancing the reliability of the research outcomes [6]. Among these techniques, summary-data-based Mendelian randomization (SMR) plays a crucial role in investigating the potential causal associations between the expression quantitative trait loci (eQTLs) of differentially expressed genes and genome-wide association study (GWAS) of disease. This method elucidates the mechanisms by which gene expression is regulated by genetic variations, which may be pivotal in the occurrence and progression of LUAD. Moreover, SMR analysis utilizes genetic polymorphisms as proxy variables to explore potential causal links between specific genes and disease development, offering robust support for identifying novel therapeutic targets [7]. By integrating multi-omics analysis with SMR analysis, researchers can more deeply explore the potential causal relationships between specific genes and diseases [8]. These methods can effectively determine whether the changes in gene expression are the cause or the consequence of the disease, thereby facilitating the identification of gene targets with significant therapeutic potential.

**Figure 1:**
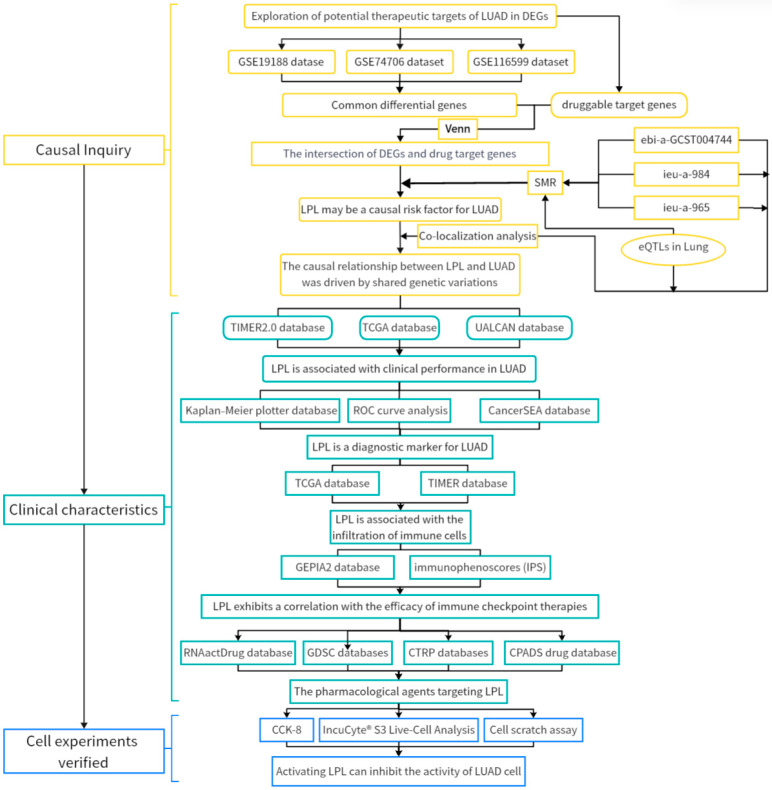
The process of identifying lipoprotein lipase (LPL) as a potential biomarker for predicting LUAD, assessing its prognosis, and evaluating the effectiveness of immune checkpoint therapy is illustrated in a flowchart. This methodology is structured into three main phases: investigation of potential causal associations, clinical feature assessment, and experimental validation using cell models.

In this research, our objective was to integrate bioinformatics analysis and cell functional experiments in order to determine potential novel therapeutic targets of considerable significance in LUAD. To achieve this, we initially analyzed the publicly accessible LUAD multi-omics RNA-seq data, identified the differentially expressed drug target genes in LUAD, and screened out the genes that could act as drug targets. Subsequently, we conducted SMR analysis to investigate the putative effects or potential causal associations of lung tissue-specific eQTLs associated with differentially expressed genes on LUAD susceptibility, leveraging genome-wide association study (GWAS) datasets. After conducting multiple comparison corrections of the *p*-values of the analysis results, we identified the genes that may be associated with an increased risk of LUAD development. Next, we explored the possible clinical relevance of these targets with respect to disease diagnosis, prognosis, immune checkpoint therapy response, and drug sensitivity. Finally, we employed several techniques, such as the Cell Counting Kit-8 (CCK-8) assay, the cell scratch wound healing assay, and the IncuCyte^®^ S3 live-cell imaging system, to assess the effects of targeted therapy on genes strongly associated with the risk of LUAD. This was carried out to evaluate the functional effects on LUAD cells and to confirm the therapeutic targets we suggested. These discoveries will contribute to deepening our understanding of the pathogenesis of LUAD and offer novel ideas for the targeted therapy of this disease. The specific workflow is presented in Figure 1.

## 2. Materials and Methods

### 2.1. LUAD Data Acquisition

The transcriptomic datasets, along with the associated clinical information of individuals diagnosed with LUAD, were retrieved from The Cancer Genome Atlas (TCGA) database (https://portal.gdc.cancer.gov/, accessed on 30 June 2024). We utilized a dataset comprising 519 LUAD tissue samples and 58 normal lung tissue samples, with sample IDs annotated based on the GRCh38/hg38 human genome reference. Furthermore, three gene expression datasets related to LUAD were obtained from the Gene Expression Omnibus (GEO) repository (https://www.ncbi.nlm.nih.gov/geo/, accessed on 30 April 2024): GSE19188 [9] (45 tumor samples and 65 normal lung samples), GSE74706 [10] (10 tumor samples and 18 normal lung samples), and GSE116959 [11] (57 tumor samples and 11 normal lung samples). Among these three GEO datasets, only GSE19188 contains patient survival data that can be used for survival analysis. The GSE74706 dataset does not provide patient survival information, and the GSE116959 dataset only includes follow-up information and does not provide specific survival data. Samples not related to lung adenocarcinoma or normal samples (including squamous cell carcinoma, large cell lung cancer, etc.) in these three datasets were excluded to avoid affecting the reliability of the experimental results.

### 2.2. Acquisition of Druggable Target Genes

The acquisition of druggable target genes was derived from the Drug-Gene Interactions and the druggable genome (DGIdb) [12] (https://www.dgidb.org/, accessed on 22 June 2024) as well as a compilation of available druggable genes identified by Finan et al. [13].

### 2.3. Extraction of DEGs in LUAD

We obtained microarray expression data for LUAD from the GEO database and conducted analyses using R to compare gene expression profiles between LUAD samples and normal controls. This approach aimed to identify differentially expressed genes (DEGs) associated with LUAD. In our analysis, we adopted an adjusted *p*-value of <0.05 and |log2FoldChange| > 1 as the criteria for identifying significant genes. Differential expression analysis was performed using the “DESeq2” package [9], classifying genes meeting the thresholds of |log2FoldChange| > 1 and an adjusted *p*-value < 0.05 as significantly differentially expressed. Furthermore, Venn diagram analysis was used to identify the common DEGs present in the GEO datasets, followed by intersection analysis with druggable target genes to identify differentially expressed druggable target genes.

### 2.4. The Identification of Druggable Target Gene eQTLs and LUAD GWAS

Recognizing the significant role of cis-eQTLs in drug development, characterized by their stronger association with druggable target genes and heightened tissue specificity, we utilized cis-eQTL data specifically derived from lung tissues in the Genotype-Tissue Expression project (GTEx) V8 eQTL dataset [14]. The GTEx V8 eQTL dataset comprises RNA sequencing samples from 15,201 individuals across 49 tissues derived from 838 post-mortem donors. This dataset provides a summary of cis-eQTL information for these 49 human tissues. To identify the most significant eQTLs as instrumental variables for SMR analysis, we utilized a genome-wide significance threshold of *p* < 5 × 10^−8^. As a result, 532 differential druggable target genes associated with eQTLs were identified. Genes linked to eQTLs were identified, and the LUAD GWAS data were retrieved from the OpenGWAS database (https://gwas.mrcieu.ac.uk/, accessed on 20 January 2024), with IDs including ebi-a-GCST004744 [15], ieu-a-984, and ieu-a-965 [16]. The ebi-a-GCST004744 dataset includes data on LUAD cases (*n* = 11,273) and controls (*n* = 55,483), encompassing a total of 7,849,324 Single Nucleotide Polymorphisms (SNPs). The ieu-a-984 dataset originates from the Transdisciplinary Research in Cancer of the Lung (TRICL) organization and involves LUAD cases (*n* = 11,245) versus controls (*n* = 54,619), containing a total of 10,345,176 SNPs. Lastly, the ieu-a-965 dataset was sourced from the International Lung Cancer Consortium (ILCCO) organization with LUAD cases (*n* = 3442) compared to controls (*n* = 14,894), comprising a total of 8,881,354 SNPs.

### 2.5. Mendelian Randomization Study Based on Summary Data

In order to explore the pleiotropic relationships between drug-related gene expression and LUAD, we employed SMR analysis. This method provides enhanced statistical accuracy compared to traditional two-sample MR approaches when using data from two large-scale population cohorts [17]. To refine the evaluation of causal association heterogeneity, Heterogeneity in Dependent Instruments (HEIDI) testing was performed, and results with *P*-HEIDI values below 0.05 were excluded. The SMR software (version 1.3.1) was utilized to perform both SMR and HEIDI assessments. To mitigate the impact of multiple comparisons, we implemented the Bonferroni correction approach for *p*-value adjustments, establishing a cutoff to maintain the False Discovery Rate (FDR) at 0.05 during the selection process. Subsequently, genes from our cohort that met the criteria of adjusted *p*-values less than 0.05 and *P*-HEIDI exceeding 0.05 were chosen for further co-localization evaluation.

### 2.6. Co-Localization Analysis

To assess the genetic overlap between LUAD and the druggable target genes, we applied Bayesian co-localization analysis utilizing eQTL data derived from lung tissue and GWAS summary statistics for LUAD through the “coloc” R package (version 5.2.3) [18]. The co-localization analysis encompassed five specific hypotheses (H0–H4): under H0, no causal variants are shared between the two traits; H1 posits a causal variant that influences gene expression but does not affect LUAD risk; H2 refers to a causal variant impacting LUAD risk without influencing gene expression; H3 suggests that distinct causal variants independently affect gene expression and LUAD risk; finally, H4 describes both traits as being driven by a single genetic variant. The extent of co-localization is quantified through the posterior probability of hypothesis 4 (PPH4). A PPH4 value above 0.8 signifies robust evidence for co-localization, those ranging from 0.5 to 0.8 denote moderate evidence, and values at or below 0.5 indicate minimal support for co-localization.

### 2.7. Pan-Cancer Analysis

Using the Cancer Single-Cell State Atlas (CancerSEA) ( http://biocc.hrbmu.edu.cn/CancerSEA/, accessed on 31 July 2024) [19], we explored the functional roles of druggable target genes in various cancer types, including their involvement in processes like apoptosis, stemness, invasion, proliferation, metastasis, angiogenesis, cell cycle regulation, hypoxia, quiescence, differentiation, inflammation, DNA damage response, and repair mechanisms. Additionally, the Tumor Immune Estimation Resource 2.0 (TIMER2.0) [20] was utilized to examine the differential expression of druggable target genes across various tumors in comparison to their corresponding normal tissues

### 2.8. TCGA Data Analysis

In the TCGA analysis, differential expression analysis was conducted using the “DESeq2” package (version 1.38.0). Genes with a |log2FoldChange| value exceeding 1 and an adjusted *p*-value less than 0.05 were considered to be significantly differentially expressed. The association between these druggable target genes and clinical characteristics, including age, histological stage, smoking history, and gender, were examined through the University of Alabama at Birmingham Cancer Data Analysis Portal (UALCAN) database (https://ualcan.path.uab.edu/analysis.html, accessed on 30 April 2024) [21]. In addition, to evaluate the relationship between LPL gene expression and smoking history as well as typical driver gene mutations (EGFR, KRAS), this study utilized the “Gene_Mutation” module in the TIMER2.0 database to conduct differential analysis of LPL expression in samples with different EGFR and KRAS mutations based on the TCGA-LUAD cohort data.

### 2.9. Survival Analysis

In order to assess the association between the expression levels of the target gene and overall survival in LUAD patients, we conducted a Kaplan–Meier survival analysis. This analysis leveraged the Kaplan–Meier Plotter database (https://kmplot.com/analysis/, accessed on 10 August 2024) [22], which provides extensive LUAD datasets comprising 1161 patients. The objective was to evaluate the relationship between the expression level of the target gene and the patient’s overall survival outcomes.

### 2.10. Diagnosis Value Analysis

To evaluate the diagnostic value of LPL in LUAD, we conducted Receiver Operating Characteristic (ROC) analysis using the pROC package (version 1.18.4) on LUAD datasets retrieved from the TCGA database [23]. The results were visualized with ggplot2 (version 3.4.0), emphasizing its predictive accuracy and significance within this context.

### 2.11. TMB and MSI Analysis

Tumor mutational burden (TMB) and microsatellite instability (MSI) analyses were conducted using the publicly available Sangerbox 2.0 platform [24] (http://vip.sangerbox.com/), which integrates multiple cancer genomic databases and standardized bioinformatics pipelines to enable efficient and reproducible pan-cancer multi-omics analysis. TMB was calculated as the total number of somatic, coding, base substitution, and indel mutations per megabase (Mb) of the genome. MSI scores were obtained based on preprocessed TCGA somatic mutation data using standardized algorithms provided by the platform to evaluate the level of instability across microsatellite regions. To explore the relationship between LPL expression and TMB/MSI, we extracted LPL expression data across multiple cancer types (pan-cancer) and stratified samples into high- and low-expression groups according to the median LPL expression level. Differences in TMB and MSI between the two groups were compared using the Wilcoxon rank-sum test, with statistical significance defined as *p* < 0.05. The results were visualized using lollipop plots to reveal the potential impact of LPL expression on genomic instability indicators across different cancer types.

### 2.12. Cell Lines

Human non-small-cell lung cancer cell line A549 (Cat#TCH-C116), NCI-H1299 (Cat#TCH-C269), PC-9 (Cat#TCH-C380), and NCI-H1975 (Cat#TCH-C276) were obtained from HyCyte (Suzhou, China). A549 and PC-9 cells were maintained in Advanced DMEM (500 mL; Cat#12491015; Gibco, Grand Island, NY, USA) supplemented with 10% fetal bovine serum (FBS; Cat#A5256701; Gibco) and 1% penicillin–streptomycin (Cat#15140148; Gibco). NCI-H1299 and NCI-H1975 cells were cultured in RPMI 1640 medium (Cat#11875093; Gibco) containing 10% FBS and 1% penicillin–streptomycin. All cells were cultured in a 37 °C incubator with 5% carbon dioxide. In this study, the A549 cells were at the 8th passage, H1299 cells at the 7th passage, PC-9 cells at the 4th passage, and H1975 cells at the 7th passage. All cells were used within a limited number of passages after thawing to ensure consistency and minimize experimental variability.

### 2.13. Wound Healing Assay

Lung cancer cells were seeded into 24-well plates at a density of 2 × 10^5^ cells per well and cultured in complete medium at 37 °C with 5% CO_2_ until they achieved full confluence. Linear wounds were created on the cells using a sterile 200 μL pipette tip. The cells were cultured in a medium containing 1% fetal bovine serum. The closure of each wound was observed at 0, 12, and 24 h.

### 2.14. Evaluation of the Effects of Activated LPL on A549 Cells Using the IncuCyte Live-Cell Imaging System

In this study, the effects of different concentrations of the LPL activator Ibrolipim on the proliferation of lung cancer cells were systematically evaluated. A549, PC-9, H1975, and H1299 cells were seeded in Corning 96-well plates (Cat#3599) and continuously monitored using the IncuCyte S3^®^ live-cell imaging system [25] (Essen Bioscience, Ann Arbor, MI, USA), beginning immediately after treatment initiation. Phase-contrast images were captured every 1 h for a total of 24 h at 10× magnification, acquiring four non-overlapping fields of view per well. Changes in cell confluence and number were analyzed to assess the dynamic effects of Ibrolipim on cell proliferation

### 2.15. Cell Viability Assay

To evaluate the effect of Ibrolipim on LUAD cell proliferation, lung cancer cell lines were exposed to different concentrations of Ibrolipim for a duration of 24 h. The assessment of cell proliferation was conducted through the CCK-8 colorimetric method. To ensure the reliability and precision of the findings, each experimental condition was independently repeated 3 to 4 times with biological replicates.

### 2.16. Quantitative Real-Time PCR (qRT-PCR)

To validate the effect of Ibrolipim on LPL mRNA expression, total RNA was extracted from A549 cells treated with different concentrations of Ibrolipim using TRIzol reagent (Invitrogen, Carlsbad, CA, USA), according to the manufacturer’s instructions. The RNA purity and concentration were assessed using a NanoDrop 2000/2000 C spectrophotometer (Thermo Fisher Scientific, Waltham, MA, USA). Subsequently, 2.0 μg of total RNA was reverse-transcribed into cDNA using Moloney murine leukemia virus reverse transcriptase (Promega, Madison, WI, USA; Cat# M1705). qRT-PCR was conducted on the ABI 7500 Real-Time PCR System (Applied Biosystems, Foster City, CA, USA) using SYBR Green PCR Master Mix. Each 20 μL reaction contained 1.0 μL of cDNA template. The relative expression of LPL was normalized to GAPDH using the 2^−ΔΔCt^ method. The primer sequences were as follows: LPL (NM_000237.3): Forward: 5′-ACAAGAGAGAACCAGACTCCAA-3′; Reverse: 5′-GCGGACACTGGGTAATGCT-3′; GAPDH: Forward: 5′-GGAGCGAGATCCCTCCAAAAT-3′; Reverse: 5′-GGCTGTTGTCATACTTCTCATGG-3′. Each reaction was performed in triplicate, and all experiments were repeated independently at least three times.

### 2.17. Transcription Factor Prediction of LPL

To investigate the regulatory mechanisms underlying LPL expression in lung adenocarcinoma (LUAD), four transcription factor prediction databases, namely HTFtarget, ENCODE, GTRD, and FIMO-JASPAR, were employed to predict potential transcription factors. Subsequently, the predicted results from these four methods were cross-referenced with the gene expression data from the LUAD-TCGA and Lung-GTEx datasets to identify critical transcription factors. The transcription factor analysis results were visualized using a Venn diagram.

### 2.18. Differential Expression and Pathway Enrichment Analyses

Transcriptomic data of LUAD were obtained from the TCGA database, specifically the RNA-seq data processed using the STAR pipeline under the TCGA-LUAD project. TPM-formatted expression matrices and corresponding clinical metadata were extracted. Normal tissue samples were removed to focus on tumor-specific expression profiles. All expression data were transformed using log2(TPM+1) prior to analysis. To explore LPL-associated transcriptomic differences, we classified LUAD samples into high- and low-expression groups based on the median expression of LPL. Differential gene expression between these two groups was then evaluated. Gene Set Enrichment Analysis (GSEA) was performed using R (version 4.2.1) and the clusterProfiler package (v4.4.4). Gene identifiers were converted using the org.Hs.eg.db package (version 3.16.0). The reference gene sets were obtained from the MSigDB collection using the msigdbr package (version 7.5.1), specifically the “c2.cp.all.v7.5.1.symbols.gmt” canonical pathways set, covering 2982 annotated pathways in Homo sapiens. After ID conversion, GSEA was conducted to evaluate the enrichment of pathways associated with ranked gene expression differences between the LPL high- and low-expression groups. The top 20 significantly enriched pathways were selected for visualization. To further investigate the biological processes associated with LPL-related differential genes, we conducted Reactome pathway enrichment analysis using the ReactomePA package (v1.49.1) in R (version 4.2.1). Prior to enrichment, gene symbols were converted to Entrez IDs using the org.Hs.eg.db package. Enrichment analysis was performed under Homo sapiens as the reference species. Pathways with adjusted *p*-values < 0.05 were considered statistically significant and used for biological interpretation.

### 2.19. Assessment of Immunotherapy Response, Immune Checkpoint Expression, and Immune Cell Infiltration

To begin with, we utilized the single-sample Gene Set Enrichment Analysis (ssGSEA) algorithm, which is incorporated in the GSVA R package [version 1.46.0] [26], to examine the relationship between LPL and immune cell infiltration across various cancers as well as in LUAD. This analysis was conducted using TCGA expression datasets and involved the use of markers that are indicative of 24 different immune cell types. We explored the association between LPL expression and the infiltration of immune cells, including T cells and B cells, in LUAD by using the TIMER database [27] (https://cistrome.shinyapps.io/timer/, accessed 20 July 2024). Additionally, using scatter plots generated from the TCGA and GTEx datasets in the Gene Expression Profiling Interactive Analysis 2 (GEPIA2) database [28] (http://gepia2.cancer-pku.cn/, accessed 10 August 2024), we examined the correlation between LPL expression and the levels of immune checkpoint genes in LUAD. To explore the association between LPL and immunophenoscores (IPSs) of PD-L1/CTLA4 in forecasting the efficacy of immunotherapy, we obtained the IPSs for LUAD cases from The Cancer Immunome Atlas (TCIA) database [29] (https://tcia.at/, accessed on 12 August 2024).

### 2.20. Single-Cell Analysis

To further assess the expression level of the target gene in tumor tissues, we employed scCancerExplorer (https://bianlab.cn/scCancerExplorer/about, accessed on 20 December 2024) [30] to analyze the single-cell data of LUAD and evaluate the expression of target genes within tumor tissues. The selected single-cell sample IDs are GSE210347, GSE117570, GSE127465, and E-MTAB-6149, all of which are from patients with non-small-cell lung cancer.2.21. Feasibility Analysis of Drug Use

We employed RNAactDrug (http://bio-bigdata.hrbmu.edu.cn/RNAactDrug/index.jsp, accessed 20 May 2024) [31] to explore potential drugs linked to the mRNA levels of target genes. Correlation analysis was carried out using the Spearman rank method. Additionally, we utilized data from the Genomics of Drug Sensitivity in Cancer (GDSC) [32] and Clinical Trial Reporting Program (CTRP) databases [33] to investigate the drug susceptibility associated with target genes. The Cancer Personalized Single-cell Atlas Data Server (CPADS) database (https://smuonco.shinyapps.io/CADSP/, accessed on 21 May 2024) [34], which is based on the guidelines for the diagnosis and treatment of LUAD released by the National Comprehensive Cancer Network (NCCN) in 2024 [35], was employed to investigate how the expression levels of target genes influence the response of LUAD to commonly used chemotherapeutic agents.

### 2.21. Statistical Analysis

Statistical analyses were conducted using GraphPad Prism 10.1.1 (GraphPad Software, San Diego, CA, USA). To assess the normality of the data distribution, we employed the Shapiro–Wilk test. Variations between the two separate cohorts were assessed through the application of *t*-tests and Mann–Whitney U tests. Each experiment was performed three times to ensure the reliability and consistency of the results. A threshold of *p* < 0.05 was considered indicative of statistical significance.

## 3. Results

### 3.1. Exploration of Potential Therapeutic Targets of LUAD in DEGs

**Figure 2:**
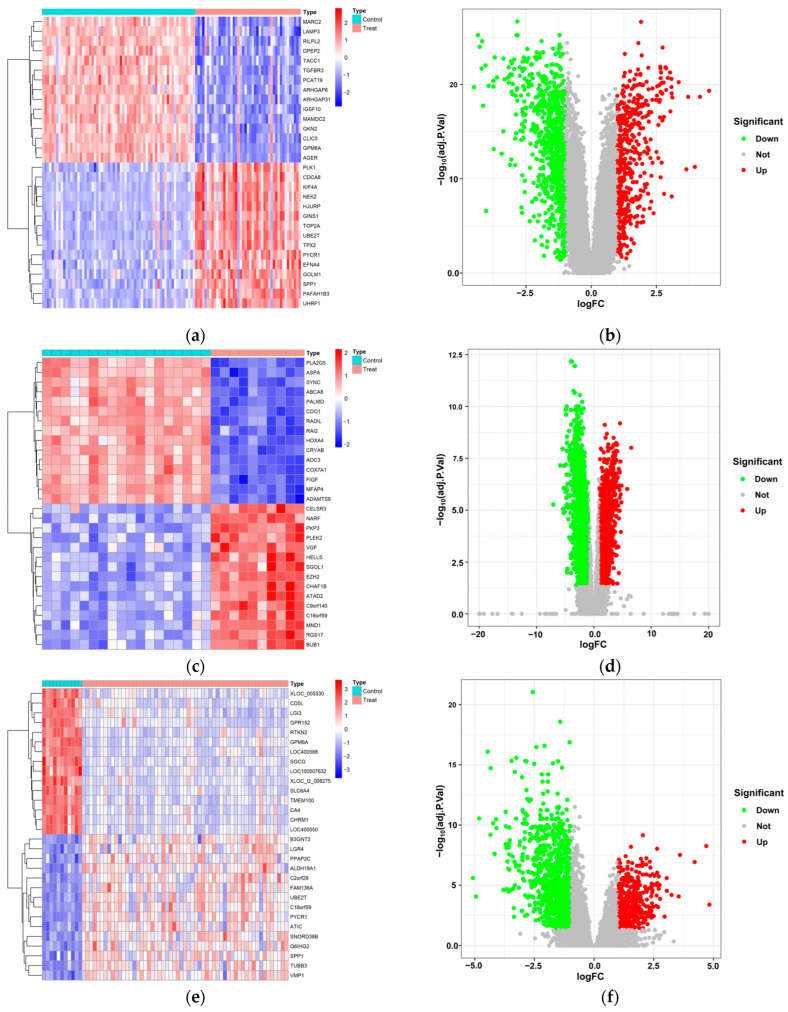

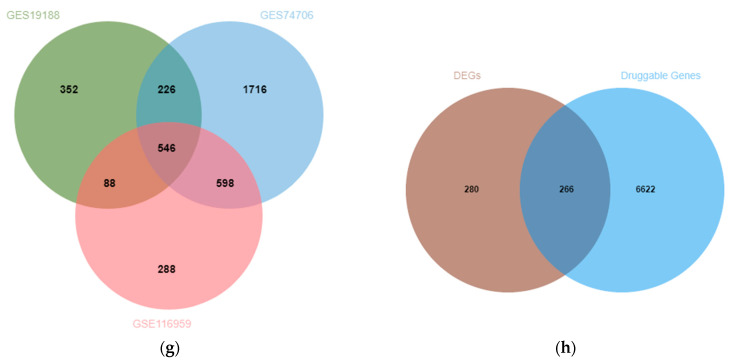
Differentially expressed druggable target genes in LUAD tissues. (**a**) The heatmap of DEGs from the GSE19188 GEO dataset for LUAD shows significant differences between cancerous and normal lung tissues. Color intensity variations reflect gene expression levels, where red denotes elevated expression and blue signifies diminished expression. (**b**) The volcano plot illustrating DEGs within the GSE19188 dataset highlights both expression alterations and their statistical significance. The *X*-axis represents the log-transformed fold change in gene expression, whereas the *Y*-axis illustrates the statistical significance of differential expression, presented as the negative logarithm of the *p*-value (−log10 *p*-value). Red points signify a need for comprehensive DEGs, whereas green points denote non-significant differential expressions. (**c**,**d**) Heatmap and volcano plot for GSE74706 dataset; (**e**,**f**) heatmap and volcano plot for GSE116,599 dataset; (**g**) shows a Venn diagram comparing DEGs across three GEO datasets; (**h**) portrays a Venn diagram illustrating overlaps between differential expressional and druggable target genes.

In order to explore potential targets of LUAD, we conducted a systematic analysis of DEGs using three LUAD datasets (GSE19188, GSE74706, and GSE116959) from the GEO database. To investigate LUAD, we analyzed gene expression patterns by comparing tumor tissues to normal lung tissues, identifying a subset of genes with significant differential expression. These DEGs exhibited marked expression differences as presented in heatmaps (Figure 2a,c,e) and volcano plots (Figure 2b,d,f), indicating molecular characteristic disparities between LUAD and normal lung tissues. To further determine the consistency of differently expressed genes across various datasets, Venn diagram analyses were performed. The results indicated that 546 common genes displayed consistent differential expression in all three datasets related to LUAD (Figure 2g). The persistent differential expression of these genes may indicate their involvement in the development and progression of LUAD, emphasizing the importance of a thorough investigation into their specific roles and the underlying mechanisms driving this disease. In order to investigate the therapeutic potential of these differentially expressed genes as potential drug targets, we intersected these 546 differential genes with a known set of 6888 druggable target genes. This intersection revealed that 266 gene candidates are both differentially expressed in LUAD and recognized as established druggable targets (Figure 2h), and may serve as promising therapeutic targets for treating LUAD.

### 3.2. LPL Is a Potential Causal Risk Factor for LUAD

**Figure 3:**
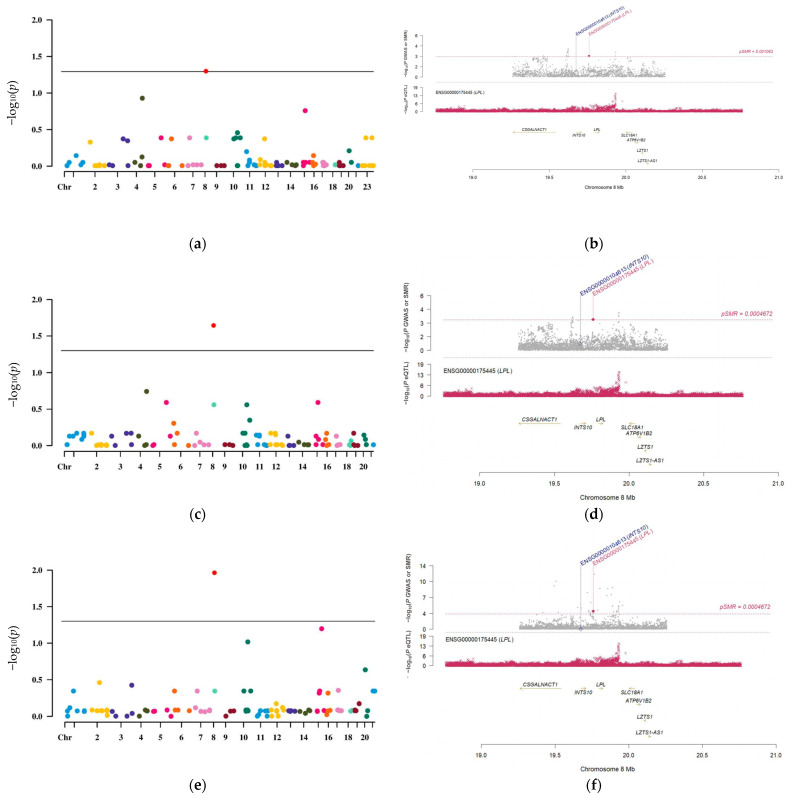
The potential causal relationship between LPL and LUAD as inferred by SMR analysis. (**a**) Manhattan plot of the SMR results for differential druggable target genes in lung tissue based on eQTLs, associated with LUAD GWAS identified by ID number ebi-a-GCST004744. The *X*-axis represents chromosome position, while the *Y*-axis displays −log (FDR) values, different colors represent genes on different chromosomes. (**c**) Manhattan plot of the SMR results for differential druggable target genes in lung tissue concerning eQTLs, linked to LUAD GWAS with ID number ieu-a-984, different colors represent genes on different chromosomes. (**e**) Manhattan plot depicting the SMR results for differential druggable target genes in lung tissue according to eQTLs from the LUAD GWAS denoted by ID number ieu-a-965, different colors represent genes on different chromosomes. (**b**,**d**,**f**) Linkage disequilibrium effect plots illustrating LPL gene associations across three different LUAD GWAS studies. These figures demonstrate a significant correlation between genetic variants of the LPL gene and risk factors associated with LUAD through linkage disequilibrium effects observed in various GWAS datasets.

To discover meaningful targets for the treatment of LUAD, we performed SMR analysis on eQTLs of druggable genes exhibiting differential expression in lung tissue, integrating these findings with LUAD GWAS data to identify candidate genes linked to LUAD development. By performing SMR analysis and comparing eQTLs of druggable target genes in lung tissue and three distinct GWAS datasets for LUAD (with IDs ebi-a-GCST004744, ieu-a-984, and ieu-a-965), we found that only the LPL gene exhibited a corrected *p*-value less than 0.05 across all three datasets (Figure 3a–f; FDR_ebi-a-GCST004744_ = 0.492631; *P*-HEIDI_ebi-a-GCST004744_ = 0.204936; FDR_ieu-a-984_ = 0.492631; *P*-HEIDI_ieu-a-984_ = 0.204936; FDR_ieu-a-965_ = 0.492631; *P*-HEIDI_ieu-a-965_ = 0.204936). These results suggest a potential causal connection between the LPL gene and the progression of LUAD. We further investigated the linkage disequilibrium effects and directional analysis concerning the LPL gene. The results indicated a notable linkage correlation between LPL’s eQTLs and those from LUAD GWAS data, and low expression levels of LPL were positively correlated with an increased risk of developing LUAD (SMR-OR [95%] ebi-a-GCST004744 = 0.78314 [0.69983, 0.91213]; SMR-OR [95%] ieu-a-984 = 0.78445 [0.68561, 0.89756]; SMR-OR [95%] ieu-a-965 = 0.66779 [0.54461, 0.81890], respectively). These findings provide preliminary evidence suggesting that LPL may be a promising therapeutic target for LUAD, highlighting its significant involvement in the development and progression of this malignancy.

### 3.3. The Causal Link Between LPL and LUAD Is Shaped by Common Genetic Variations

**Figure 4:**
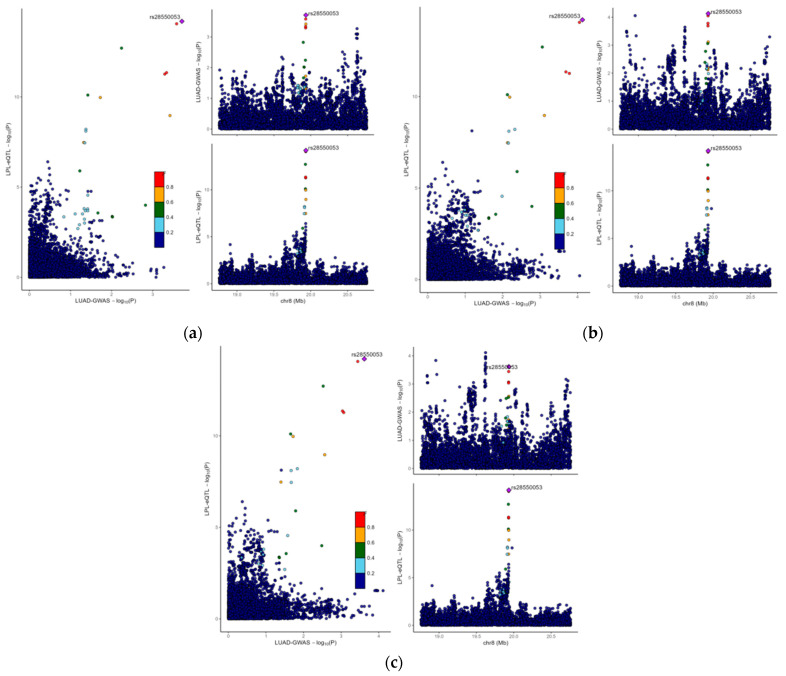
The potential causal relationship between LPL and LUAD was driven by shared genetic variations. (**a**) Co-localization analysis comparing the eQTL of LPL in lung tissue and the GWAS for LUAD (ebi-a-GCST004744). This figure illustrates the co-localization of the eQTL for the LPL gene with signals from the LUAD GWAS on chromosomes, with a PPH4 value exceeding 0.9, suggesting that both share a common genetic signal. (**b**) Co-localization analysis comparing the eQTL of LPL in lung tissue and the GWAS for LUAD (ieu-a-984). (**c**) Co-localization analysis comparing the eQTL of LPL in lung tissue and the GWAS for LUAD (ieu-a-965).

To further confirm the causal connection between the LPL gene and LUAD, we performed co-localization analysis of the LPL gene. This approach evaluates whether the eQTLs of LPL exhibit overlapping genetic signals with those identified in LUAD GWAS. The comparison of the eQTLs of the LPL gene in lung tissue with three LUAD GWAS datasets (IDs: ebi-a-GCST004744, ieu-a-984, and ieu-a-965) showed a high level of co-localization signal for the LPL gene’s eQTLs with LUAD GWAS across all three datasets, with PPH4 values exceeding 0.8 (Figure 4a–c; PPH4ebi-a-GCST004744 = 0.805; PPH4ieu-a-984 = 0.931; PPH4ieu-a-965 = 0.900). This finding suggests a potential causal role for the LPL gene in LUAD, and the high PPH4 values suggest that within the same region, the eQTLs of the LPL gene and GWAS signals for LUAD share overlapping genetic variants. This enhances the credibility of considering LPL as a potential druggable target for LUAD but also lays a foundation for future research into specific mechanisms by which LPL contributes to its pathogenesis.

### 3.4. LPL Is Associated with Clinical Performance in LUAD

**Figure 5:**
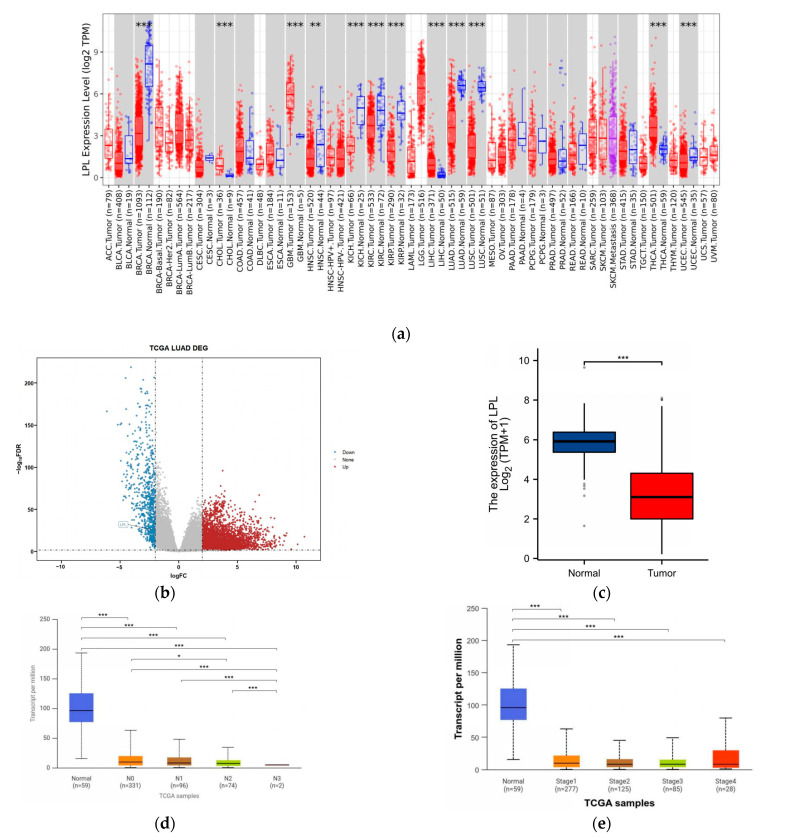

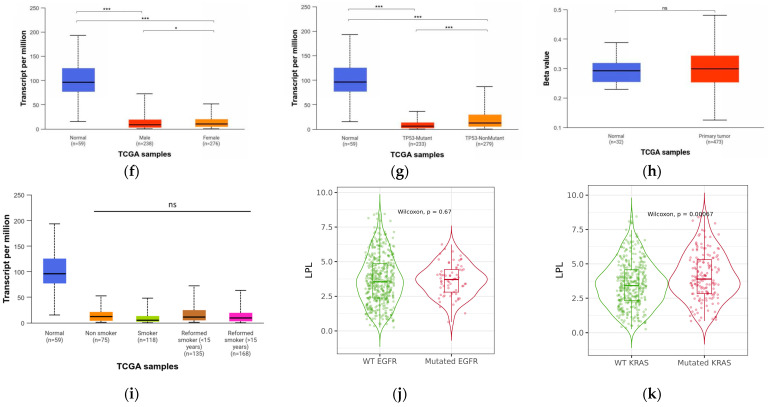
Illustration of the expression of LPL across various tumors, highlighting its notably low expression in LUAD, which correlates with several clinical characteristics. (**a**) The significant *p*-value for LPL in LUAD from the TCGA database, as provided by the TIMER2.0 database, suggests a notable difference in LPL expression between tumor tissue and adjacent normal tissue across all tumors in the TCGA dataset; (**b**) analysis of all DEGs within the LUAD TCGA data reveals that LPL has significantly downregulated; (**c**) the mRNA expression of LPL in the LUAD cohort from the TCGA database; (**d**) investigation into the differences in LPL expression across various stages of lymph node metastasis associated with LUAD; (**e**) analysis of variations in LPL expression at different stages of LUAD; (**f**) examination of LPL expression differences based on patient sex among individuals diagnosed with LUAD; (**g**) assessment of discrepancies in LPL expression among patients harboring TP53 mutations within cases of LUAD; (**h**) evaluation of mRNA methylation levels affecting LPL expression among patients diagnosed with LUAD; (**i**) the expression differences of LPL among patients with different smoking histories in the TCGA dataset; (**j**) the expression differences of LPL among different EGFR mutation groups; (**k**) the expression differences of LPL among different KRAS mutation groups. * *p* < 0.05, ** *p* < 0.01, *** *p* < 0.001, ns > 0.05.

We further explored the expression patterns and clinical characteristics of LPL in LUAD and various other cancers. Meanwhile, previous studies have demonstrated that LPL expression is decreased in NSCLC through IF or IHC techniques [36], while it is significantly overexpressed in gastric cancer [37]. Reanalysis leveraging the TIMER2.0 database (Figure 5a) demonstrated substantial variations in LPL gene expression between tumor tissues and their corresponding normal counterparts across multiple tumor types, with significantly reduced LPL expression levels identified specifically in LUAD tissues. This suggests that LPL may play a vital role in LUAD onset and advancement. Whole-genome differential expression analysis conducted using the Cancer Genome Atlas (TCGA) database indicated that LPL is one of the significantly downregulated genes in LUAD tissues (Figure 5b,c). This finding suggests that the reduced expression of LPL’s eQTLs may act as a protective factor against the risk of LUAD. Subsequently, we explored variations in mRNA expression of the LPL gene concerning histological staging, gender, lymph node metastasis, TP53 mutations, and methylation through the UALCAN database. The findings revealed notable variations in LPL mRNA expression across different stages of lymph node metastasis. Specifically, LPL levels at the N3 stage were considerably lower compared to those at the N0, N1, and N2 stages (Figure 5d). Importantly, no significant differences were observed among patients with varying histological stages (Figure 5e). Additionally, male patients exhibited significantly lower LPL mRNA levels compared to female patients (Figure 5f). Moreover, an analysis of TP53 mutations indicated that patients with TP53 mutations had markedly reduced LPL expression relative to those without these mutations (Figure 5g). Furthermore, comparative analysis revealed no statistically distinct methylation profiles at the LPL locus between controls and LUAD cohorts (Figure 5h), implying that the elevated risk of LUAD linked to low LPL expression is not due to changes in its methylation status. Meanwhile, comparative analysis revealed that there was no statistically significant difference in LPL expression among patient samples with different smoking histories (Figure 5i), indicating that LPL expression is not significantly associated with the smoking history of the patients. Mutation correlation analysis indicated that the expression of LPL was not associated with different EGFR mutation samples (Figure 5j), but was significantly increased in KRAS mutation samples (Figure 5k).

### 3.5. LPL Is a Diagnostic Marker for LUAD

**Figure 6:**
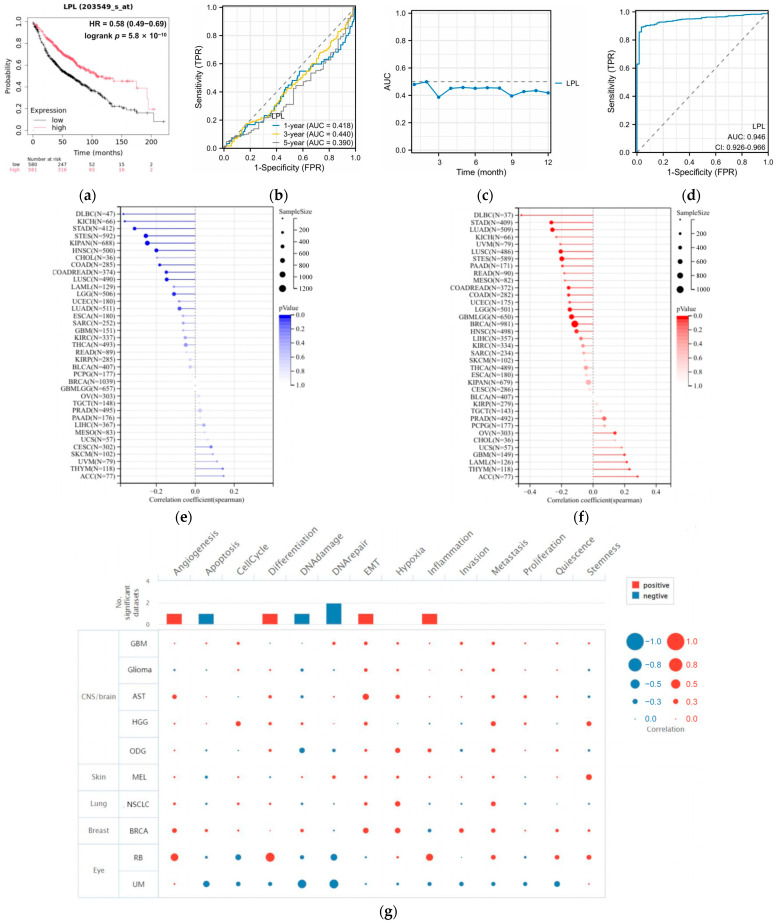
The diagnostic and prognostic value of LPL in LUAD. (**a**) Survival difference analysis of LPL expression between LUAD patients and normal individuals; (**b**) ROC curve analysis of LPL at different stages of development in LUAD patients, the dotted line indicates that the AUC is equal to 0.5; (**c**) the AUC for LPL over a one-year follow-up period in LUAD patients, the dotted line indicates that the AUC is equal to 0.5; (**d**) the contribution of LPL to the diagnosis of LUAD, the dotted line indicates that the AUC is equal to 0.5; (**e**) results of MSI analysis for LPL in multiple cancers; (**f**) results of TMB analysis for LPL in multiple cancers; (**g**) the functional roles of LPL and its links to 14 distinct cancer types, as documented in the CancerSEA database, were examined.

We assessed the diagnostic and prognostic value of LPL for LUAD. To gain deeper insight into the prognostic significance of LPL in individuals with LUAD, survival analysis utilizing the Kaplan–Meier plotter database revealed that elevated LPL expression may be associated with improved patient outcomes (Figure 6a). To further assess the predictive value of LPL for patient survival outcomes, we generated time-dependent ROC curves at various time points (1 year, 2 years, and 5 years) (Figure 6b,c). The results indicated that the AUC values of LPL at each of these time intervals were all below 0.5, suggesting that the effectiveness of LPL in predicting the prognosis of LUAD is limited. We next assessed the potential diagnostic value of LPL for LUAD and found an AUC of 0.946 (Figure 6d), suggesting significant diagnostic potential for LPL. As LPL has a good diagnostic value for LUAD, we analyzed the correlation between LPL and the commonly used clinical immune markers of LUAD (TTF1, CK7, NAPSA). The results showed (Figure S1) that there were significant differences in the expression of LPL and TTF1, CK7 [38,39,40], and NAPSA (*p* < 0.05), but the correlations between LPL and these three immune markers were not strong (the absolute values of R were all around 0.1). As LPL is associated with poor prognosis in LUAD, we conducted a further analysis of the role of LPL in LUAD. The microsatellite instability (MSI) analysis results for LPL showed a significant negative correlation with MSI in 10 types of cancer including LGG, COAD, and COADREAD (Figure 6e). Additionally, the tumor mutational burden (TMB) analysis results for LPL indicated a significant positive correlation with TMB in five types of cancer, namely GBM, LAML, THYM, OV, and ACC (*p* < 0.05), but a negative correlation with TMB in thirteen types of cancer including GBMLGG, LGG, LUAD, COAD, and COADREAD (Figure 6f). To explore why LPL could be used as a diagnostic marker for LUAD, we utilized data from CancerSEA for functional state analysis (Figure 6g) and identified a potential relationship between LPL and several key biological processes associated with NSCLC, such as negative correlations with angiogenesis, cell cycle progression, differentiation, epithelial–mesenchymal transition, hypoxia, and metastasis; on the other hand, positive correlations were observed with apoptosis, DNA damage response, DNA repair mechanisms, inflammation, invasion capabilities, proliferation rate, quiescence states, and stemness characteristics. These biological mechanisms are pivotal in influencing tumor invasiveness and metastatic potential, further indicating that LPL could be a significant contributor to the progression of malignant phenotypes in LUAD. TTF1 is a highly valuable biomarker for the diagnosis of LUAD. Exploring the correlation between LPL and TTF1 gene expression has a certain reference value for verifying the diagnostic value of LPL. Gene correlation analysis showed no significant correlation between the expression of LPL and TTF1 in LUAD.

### 3.6. The LPL Activator Ibrolipim Inhibits the Proliferation and Migration of LUAD Cells

**Figure 7:**
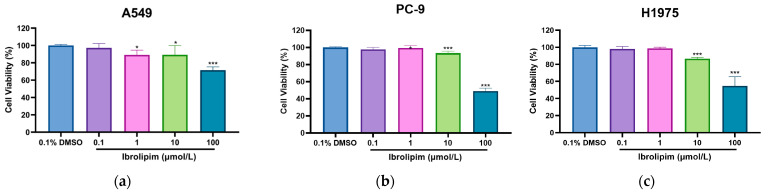

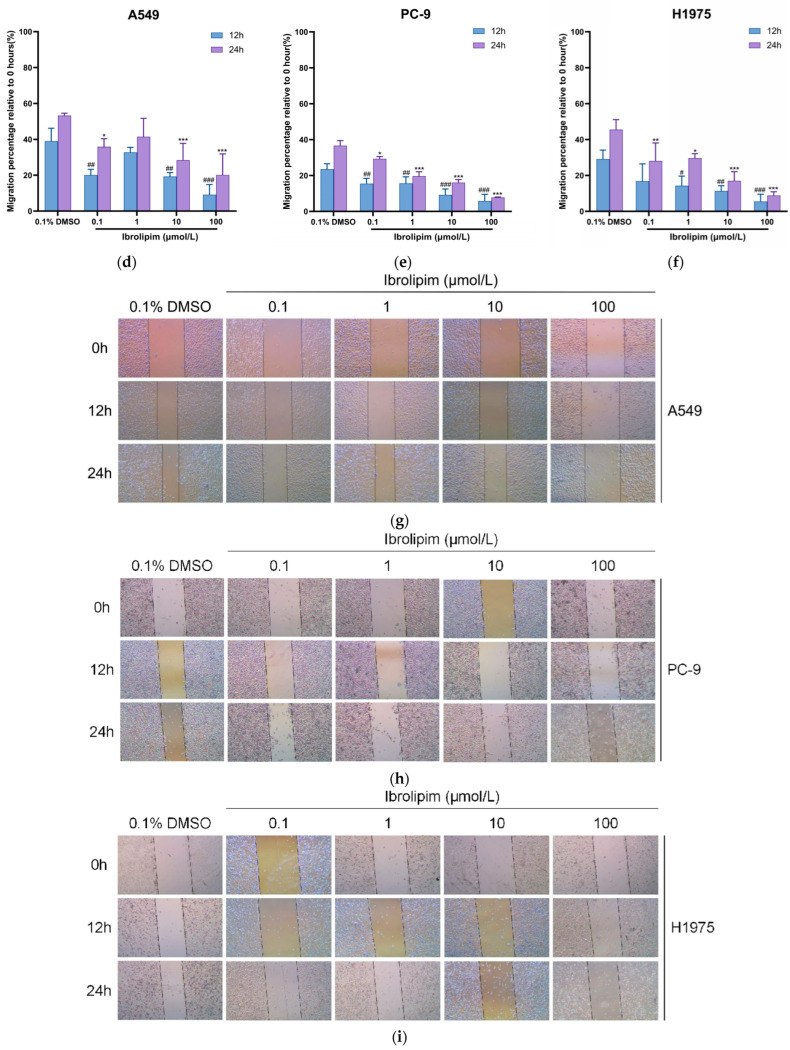
The LPL activator Ibrolipim has the ability to suppress both the proliferative capacity and migratory potential of LUAD cells. (**a**) The viability of A549 cells was assessed after treatment with varying concentrations of Ibrolipim for 24 h; (**b**) the viability of PC-9 cells was assessed after treatment with varying concentrations of Ibrolipim for 24 h; (**c**) the viability of H1975 cells was assessed after treatment with varying concentrations of Ibrolipim for 24 h (* indicates significant differences between each dose group and the Control group. * *p* < 0.05, ** *p* < 0.01, *** *p* < 0.001); (**d**) comparison of migration rates of A549 cells in different treatment groups; (**e**) comparison of migration rates of PC-9 cells in different treatment groups; (**f**) comparison of migration rates of H1975 cells in different treatment groups (# indicates significant differences between each dose group and the Control group at 12 h, * indicates significant differences between each dose group and the Control group at 24 h. * *p* < 0.05, ** *p* < 0.01, *** *p* < 0.001; # *p* < 0.05, ## *p* < 0.01, ### *p* < 0.001); (**g**) migration analysis of A549 cells after treatment with varying doses of Ibrolipim for 24 h; (**h**) migration analysis of PC-9 cells following exposure to different concentrations of Ibrolipim for 24 h; (**i**) migration analysis of H1975 cells following exposure to different concentrations of Ibrolipim for 24 h.

Due to the potential causal relationship between LPL and LUAD as well as its diagnostic value, herein, we further investigated the anti-tumor effect of enhancing the expression and activity of LPL. Ibrolipim functions as an enhancer of LPL activity [41]. Previous studies have documented that Ibrolipim is capable of significantly increasing the expression level and activity of LPL [42,43] and it is often used in clinical practice to improve lipid metabolism. Therefore, in this study, different doses of Ibrolipim (0.1, 1, 10, and 100 μmol/L, dissolved in 0.1% dimethyl sulfoxide) were used to treat LUAD cells (A549 cells, PC-9 cells, and H1975 cells) to activate LPL activity and enhance the expression of LPL-related mRNA in LUAD cells, simulating the increase in LPL mRNA expression in the lung tissues of LUAD patients. The cell viability of LUAD cells lines (A549, PC-9, and H1975) after the administration of the LPL activator Ibrolipim was observed through in vitro cell experiments such as CCK8, cell scratch, and the IncuCyte live-cell imaging system. The findings from the cell proliferation activity CCK-8 assay (Figure 7a–c) indicated that the multiplication activity of LUAD cells was suppressed following treatment with Ibrolipim, especially at a treatment dose of 100 μmol/L, where the activities of A549, PC-9, and H1975 cells were significantly affected (*p* < 0.001). The cell scratch assay results (Figure 7d–h) indicated that the migration capability of A549, PC-9, and H1975 cells was substantially suppressed at a treatment concentration of 100 μmol/L (*p* < 0.001). At the same time, when A549 cells were treated with another activator of LPL, apoC II, a notable suppression of cell proliferation was observed at a concentration of 1 μg/mL (Figure S2a). Additionally, we observed that treatment with Ibrolipim could also significantly inhibit the cell viability and migration ability of H1299 cells (Figure S3a,b,d), suggesting that LPL may also have certain value in large-cell lung cancer.

**Figure 8:**
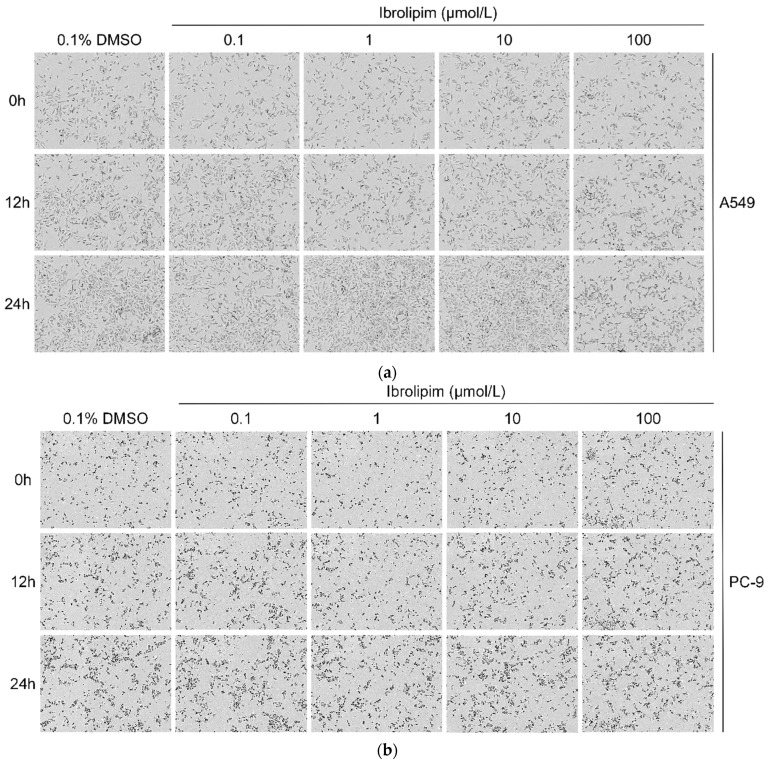

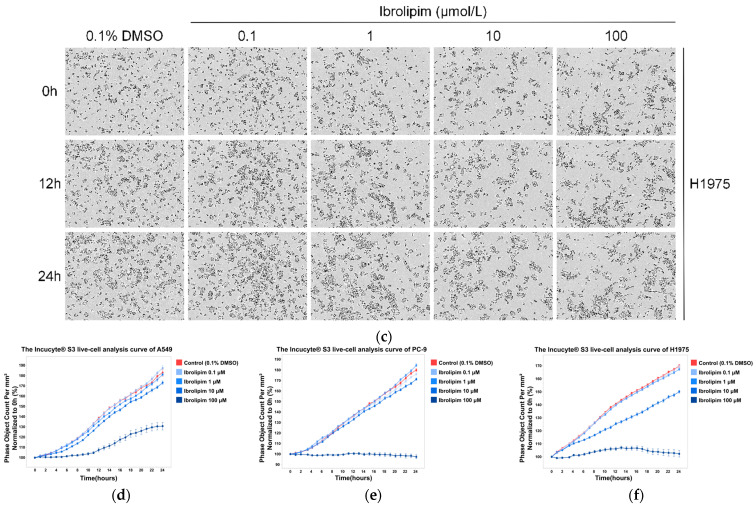
The proliferation of A549 and H1299 cell lines treated with Ibrolipim was observed using the IncuCyte^®^ S3 live-cell analysis system within 0 to 24 h. (**a**) The observation results of live cells in each group of the A549 cell line at 0, 12, and 24 h; (**b**) the observation results of live cells in each group of the PC-9 cell line at 0, 12, and 24 h; (**c**) the observation results of live cells in each group of the H1975 cell line at 0, 12, and 24 h; (**d**) the relative change curve of cell area in each group of the A549 cell line; (**e**) the relative change curve of cell area in each group of the PC-9 cell line; (**f**) the relative change curve of cell area in each group of the H1975 cell line.

The IncuCyte^®^ S3 live-cell analysis system is a live-cell imaging platform that facilitates the real-time observation, analysis, and quantification of biological processes in living cells using time-lapse microscopy [44]. IncuCyte^®^ S3 helps assess cell health, such as proliferation, cytotoxicity, and apoptosis. The advantages of IncuCyte^®^ S3 include simple sample preparation, the ability to select specific time points for image acquisition, the automated configuration of cell parameters for monitoring, and real-time graphical representation. In our study, the IncuCyte^®^ S3 live-cell analysis system was used to observe the changes in cell number after the administration of Ibrolipim [45]. After normalizing the cell counts at each time point relative to the initial time point for each group, statistical analysis was performed using Prism to generate the results presented in Figure 8. The results indicated that treatment with 100 μmol/L Ibrolipim could inhibit the proliferation of A549 cells (Figure 8a,d), PC-9 cells (Figure 8b,e), and H1975 cells (Figure 8c,f). Meanwhile, treating A549 cells with 1 μg/mL apoC II could also conspicuously inhibit the proliferation of the cells (Figure S2b,c). Similarly, using the IncuCyte^®^ S3 live-cell analysis system, we observed that Ibrolipim significantly suppressed the proliferative activity of H1299 cells, also suggesting the important role of LPL in non-small-cell lung cancer (Figure S3c,e).

### 3.7. The Possible Regulatory and Functional Mechanisms of LPL in Lung Adenocarcinoma

**Figure 9:**
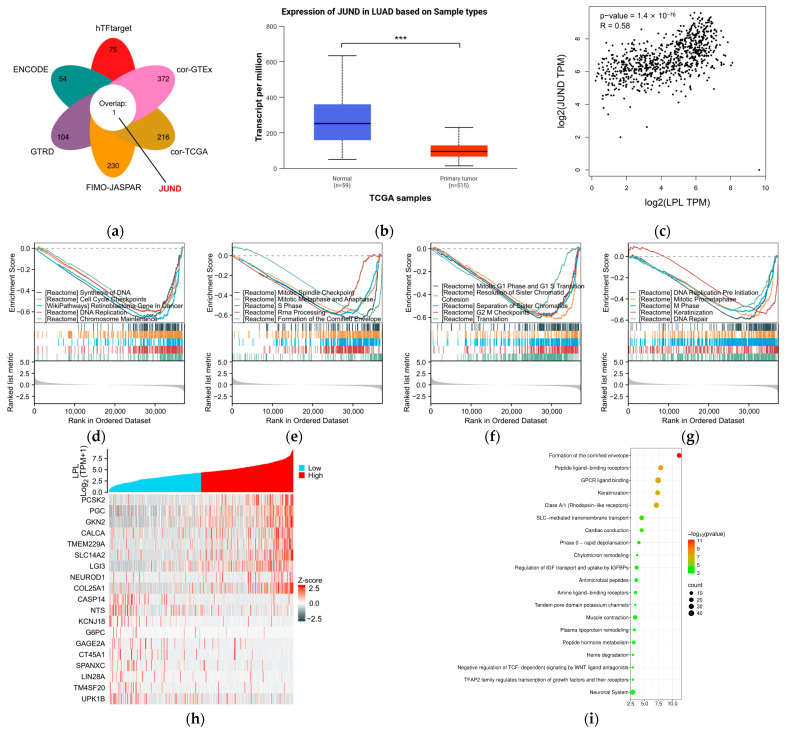
The upstream regulatory mechanism and downstream action pathways of LPL in LUAD were predicted. (**a**) One transcription factor was predicted through four databases in conjunction with the LUAD-TCGA and Lung-GTEx datasets; (**b**) the expression level of the JUND transcription factor was verified in the LUAD-TCGA dataset (*** *p* < 0.001); (**c**) correlation analysis between JUND and LPL in LUAD; (**d**) the top 5 significantly enriched pathways in the GSEA analysis of LPL; (**e**) the 6th- to 10th-most significantly enriched pathways in the GSEA analysis of LPL; (**f**) the 11th- to 15th-most significantly enriched pathways in the GSEA analysis of LPL; (**g**) the 16th- to 20th-most significantly enriched pathways in the GSEA analysis of LPL; (**h**) heatmap of genes significantly associated with LPL (the top 10 negatively correlated and the top 10 positively correlated); (**i**) Reactome pathway enrichment results of all differentially expressed genes.

To verify whether Ibrolipim treatment could upregulate LPL expression at the transcriptional level, quantitative real-time PCR was performed on A549 cells treated with different concentrations of Ibrolipim (0, 1, 10, and 100 μmol/L). The results showed that compared with the untreated control group, 100 μmol/L Ibrolipim significantly increased LPL mRNA expression in A549 cells (Figure S4). Additionally, as the cell experiments showed that the LPL inhibitor Ibrolipim can inhibit the proliferation and migration of LUAD A549, PC-9, and H975 cells, in this part, we will further explore the potential regulatory and tumor-influencing mechanisms of LPL in LUAD. To explore the upstream mechanism of LPL expression dysregulation in LUAD, we conducted a predictive analysis of potential transcription factors (TFs) related to LPL expression. By integrating the results from four transcription factor databases and TCGA data and GETx lung data for LUAD, we identified one potential transcription factor (JUND) (Figure 9a). Further differential analysis revealed that JUND expression was significantly decreased in LUAD (Figure 9b; *p* < 0.05), and it was significantly positively correlated with LPL expression in LUAD (Figure 9c; *p* < 0.05, R > 0). Therefore, the JUND transcription factor may regulate the expression of the LPL gene and affect its transcriptional level. We further investigated the signaling pathways associated with LPL in LUAD. First, using GSEA, we identified pathways differentially enriched between the high- and low-LPL-expression groups. The results revealed that LPL expression in LUAD is closely associated with multiple critical biological processes, including DNA synthesis, cell cycle checkpoints, DNA replication, chromosome maintenance, and mitotic spindle checkpoint regulation (Figure 9d–g; the results of each pathway analyzed by GSEA are presented separately in Figure S5). Subsequently, we divided the LUAD samples from the TCGA dataset into high- and low-LPL-expression groups and identified significantly differentially expressed genes (DEGs) associated with LPL expression. Representative DEGs were visualized in a heatmap (Figure 9h). We then performed Reactome pathway enrichment analysis on all LPL-associated DEGs to predict the potential signaling pathways involved (Figure 9i). The analysis identified several enriched pathways, including formation of the cornified envelope, keratinization, phase 0—rapid depolarization, chylomicron remodeling, and plasma lipoprotein remodeling, suggesting that LPL may participate in both metabolic and epithelial differentiation-related processes in LUAD.

### 3.8. LPL Is Associated with the Infiltration of Immune Cells in LUAD

**Figure 10:**
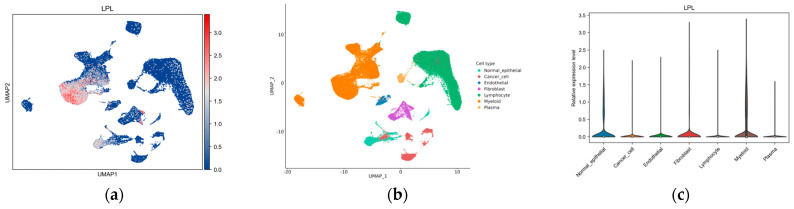

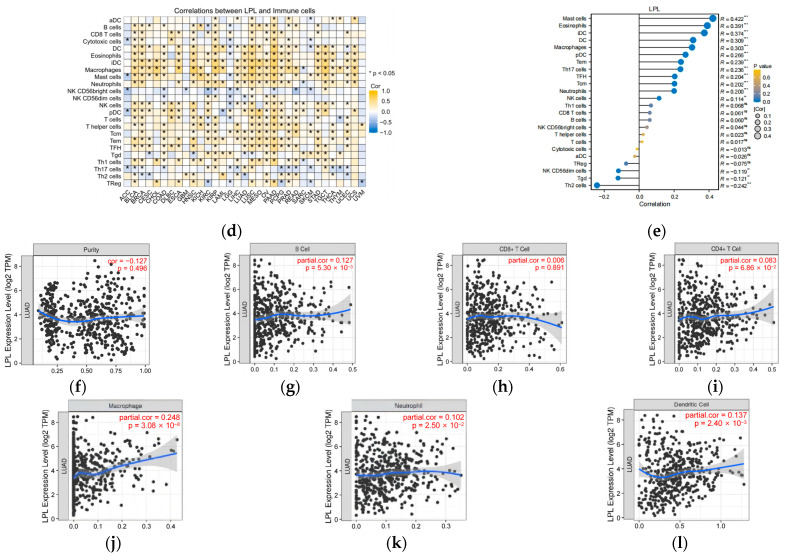
Correlation between LPL and immune cells. (**a**) The relative expression levels of LPL in GSE210347; (**b**) the clustering situation of single-cell results in GSE210347; (**c**) violin plot of LPL expression in relevant cells in GSE210347; (**d**) immune infiltration heatmap of LPL across various cancers in TCGA; (**e**) lollipop chart depicting immune infiltration of LPL in LUAD from TCGA (** *p* < 0.01, *** *p* < 0.001, ns > 0.05); (**f**–**l**) bivariate scatterplots demonstrating correlations between LPL transcriptional activity and immunological profiling parameters, including B lymphocytes, T cell subsets (CD4+/CD8+), granulocytes (neutrophils), and antigen-presenting cells (macrophages, dendritic cells).

We further evaluated the immune correlation between LPL and LUAD. We employed the scCancerExplorer database to analyze single-cell data and evaluate the expression profiles of the LPL gene in non-small-cell lung cancer as well as in adjacent normal tissues. Our investigation showed that LPL gene expression was diminished in tumor cells, whereas it was significantly elevated in tumor-infiltrating myeloid cells (Figure 10a–c and Figure S6). At the same time, utilizing information extracted from the TCGA repository, we investigated the immunological landscape linked to LPL across multiple malignancies, with a particular focus on LUAD, and examined its potential roles in immune modulation. Our analysis revealed a strong correlation between LPL expression and the presence of various immune cell types infiltrating multiple cancer types (Figure 10d). Specifically, LPL is significantly positively correlated with mast cells, monocyte macrophages, dendritic cells (DCs), and immature dendritic cells (iDCs), while showing a significant negative correlation with natural killer (NK) cells. The results suggest that LPL could play an intricate role in regulating immune interactions within the tumor microenvironment. Additional analyses of the immune infiltration characteristics of LPL in LUAD (Figure 10e) demonstrated a strong positive correlation between LPL and mast cells, DCs, iDCs, and monocyte macrophages within LUAD tissues; conversely, they demonstrated significant negative correlations with Th2 cells and γδ T cells (Tgd). These findings illuminate differential regulatory effects exerted by LPL among various types of immune cell which might influence tumor immunity evasion and local immune environment formation. A correlation analysis between LPL and various immune cell infiltrates (Figure 10f–l) revealed distinct positive associations with macrophages, B cells, CD8+ T cells, CD4+ T cells, dendritic cells, and neutrophils in LUAD. Particularly noteworthy were the prominent positive correlations observed between LPL and B cells or neutrophils alongside macrophages or dendritic cell populations; these support the potential implications for LPL’s role in modulating tumor-associated immuno-microenvironments.

### 3.9. LPL Exhibits a Correlation with the Efficacy of Immune Checkpoint Therapies

**Figure 11:**
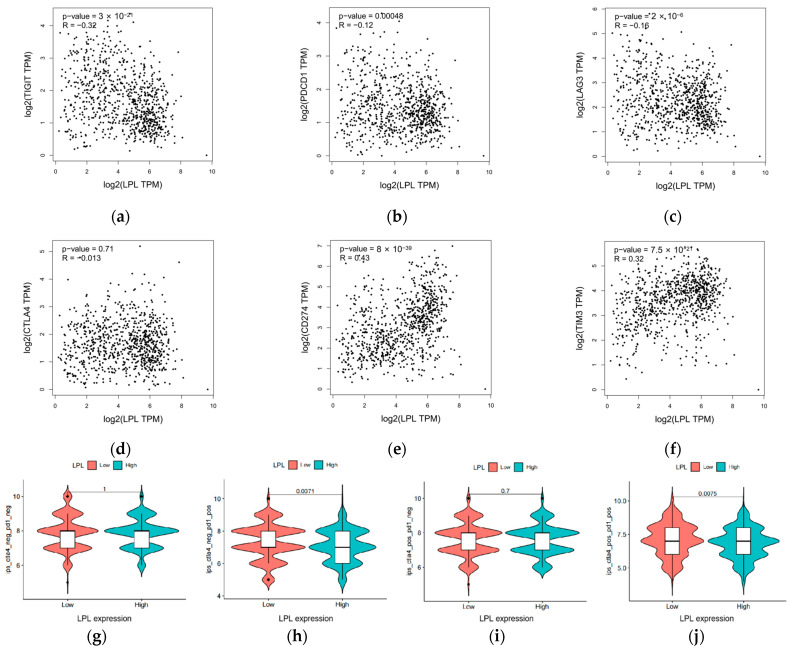
Association between LPL and immune checkpoints, along with immunotherapy response. (**a**–**f**) Scatter plots demonstrating the relationship between LPL expression and key immune checkpoint molecules, including PD-L1, TIGIT, LAG3, CTLA4, TIM3, and PD-1; (**g**–**j**) illustrate the impact of LPL on sensitivity to immune checkpoint blockade therapies.

LPL is associated with immune infiltration in various immune cells in LUAD. Here, we further evaluated the value of LPL for immune checkpoint therapy in LUAD. We examined the relationship between LPL and immune checkpoint molecules and found that key immune checkpoint genes, including TIGIT, PDCD1, LAG3, CD274, and CTLA4, demonstrated statistically significant correlations with LPL (*p* < 0.05), all exhibiting negative correlation coefficients (R-values) (Figure 11a–f). Given the observed inverse association between LPL expression levels and immune checkpoints, including PD-1, we explored the potential link between immune checkpoint inhibitors (ICIs) and LPL. Analysis of the IPSs of patients categorized by LPL expression levels revealed that individuals with low LPL expression displayed increased IPSs for anti-PD-1 immunotherapy, indicating a potentially improved response to this treatment in the subgroup (Figure 11g–j).

### 3.10. The Pharmacological Agents Targeting LPL

**Figure 12:**
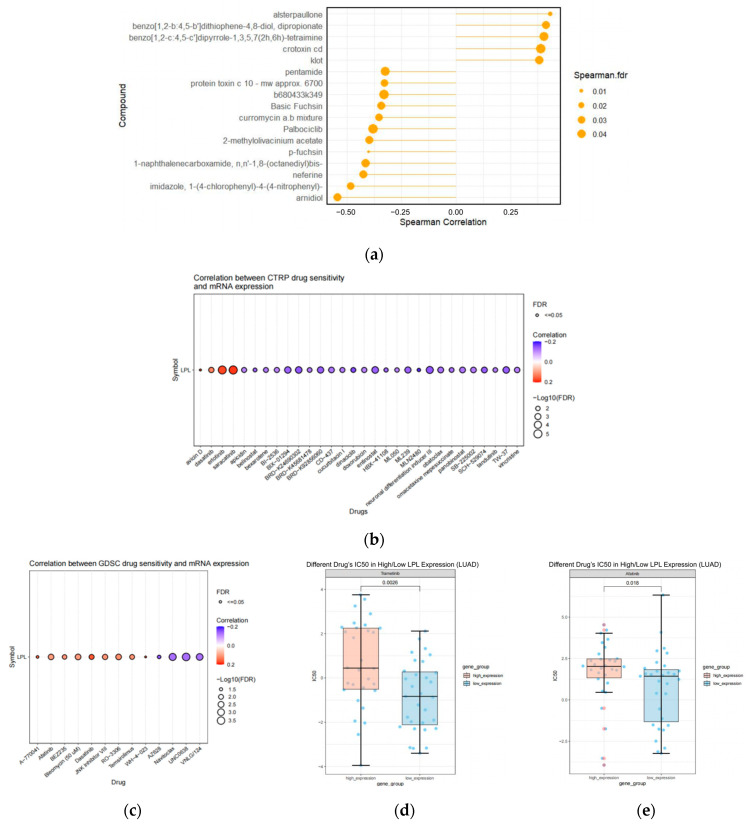
Analysis of drug-related correlations with LPL. (**a**) The lollipop plot demonstrates the Spearman correlation between LPL expression and mRNA-regulating drugs retrieved from the RNAactDrug database (FDR < 0.05, stat > 0.3); (**b**) correlation between LPL and drug sensitivity in cancer genomics as defined by Genomics of GDSC; (**c**) correlation between LPL and drug sensitivity according to the CTRP; (**d**,**e**) box plots demonstrating the antagonistic effects of trametinib and afatinib on LPL.

As LPL has a potential causal relationship with LUAD, we explored pharmacological agents that affect LPL expression, and which may be used for LUAD treatment. We explored drugs in the RNAactDrug database that correlated with LPL mRNA expression (Figure 12a). Of all the drugs with notable associations, twelve demonstrated a marked inverse relationship between their sensitivity and the expression levels of LPL mRNA (FDR < 0.05; stat < −0.3), whereas five showed a significant positive correlation (FDR < 0.05; stat > 0.3). Next, we performed an analysis of LPL mRNA expression and its responsiveness to different chemotherapy drugs by utilizing the GDSC and CTRP databases. The findings revealed that in the GDSC database, LPL expression exhibited a positive correlation with treatments including A-770041, Afatinib, BEZ235, Bleomycin, Dasatinib, JNK Inhibitor VIII, RO-3306, Temsirolimus, and WH-4-023. Conversely, it showed a negative correlation with treatments such as AZ628, Navitoclax, UNC0638, and VNLG/124 (Figure 12b). In the analysis of the CTRP database, it was found that LPL expression exhibited a positive correlation with treatments including avicin D, dasatinib, erlotinib, and saracatinib. In contrast, it demonstrated a negative correlation with several drugs including apicidin, belinostat, bexarotene, BI-2536, BIX-01294, BRD-K24690302, BRD-K45681478, BRD-92856060, CD-437, cucurbitacin I, dinaciclib, doxorubicin, and entinostat, among others (Figure 12c). Additionally, we screened potential targeted therapies for patients with low levels of LPL expression in the CPADS drug database based on the treatment guidelines from NCCN. If the half-maximal inhibitory concentration values for low-expression groups were lower than those of high-expression groups, this would imply enhanced effectiveness of chemotherapeutic agents on inhibiting low expressions of LPL. Through an investigation into agents such as paclitaxel, docetaxel, pirarubicin, gemcitabine, vincristine, erlotinib, pertuzumab, and afatinib, among others mentioned within NCCN guidelines, we identified that trametinib and afatinib could serve as potential therapeutics targeting low expressions of LPL (*p* < 0.05) (Figure 12d–e). The findings suggest that LPL functions as an indicator for predicting both cancer immunotherapy responses and the efficacy of small-molecule drugs targeting this protein, providing strong evidence to further lung cancer treatment research.

## 4. Discussion

LUAD, the most common form of lung cancer, remains one of the leading contributors to cancer mortality on a global scale [35,46]. Although there has been considerable advancement in molecular targeted therapy and immunotherapy in recent years, the prognosis for LUAD continues to be unfavorable because of its complex molecular biological features, and the five-year survival rate for some patients remains restricted [47,48]. Therefore, identifying molecular targets associated with the onset and progression of LUAD and exploring new therapeutic avenues present considerable challenges [49,50]. In this research, we detected 266 differentially expressed, druggable target genes in the lung tissues of LUAD patients via bioinformatics analysis. Additionally, we applied the SMR method to investigate the possible potential causal link between these genes and the vulnerability to LUAD. Among the numerous differentially expressed druggable target genes, we found that lower expression levels of LPL eQTLs in lung tissue may be associated with an increased risk of developing LUAD. LPL plays a crucial role as an essential enzyme in lipid metabolism; it is encoded by the LPL gene and expressed in various tissues such as the heart, lungs, muscles, and others [51,52]. This enzyme possesses dual functions as both a triglyceride hydrolase and a ligand/bridging factor. Mutations of significant severity can cause LPL deficiency, potentially leading to Type I Hyperlipoproteinemia, whereas less critical mutations may be implicated in diverse abnormalities in lipoprotein metabolism [53,54].

By integrating SMR and bioinformatics approaches, in this study, we systematically characterized the expression profile of the LPL gene in LUAD and highlighted its potential as both a druggable target and an immunomodulatory factor. A thorough examination of the LUAD dataset from TCGA showed that decreased LPL expression was associated with distinct molecular characteristics of LUAD tumor tissues. These findings not only underscored the critical role of LPL in LUAD pathogenesis but also provided a robust theoretical basis for its application as a therapeutic target in personalized treatment strategies. Regarding immune regulation, our study revealed a robust correlation between LPL expression and the presence of various immune cell populations within the tumor microenvironment. At the multi-cancer type level, the correlation between LPL expression levels and TMB and MSI reveals its potential role in tumor genomic instability. Specifically, LPL is significantly negatively correlated with MSI in 10 cancer types including LGG, COAD, and COADREAD, suggesting that LPL may be involved in maintaining microsatellite stability or regulating related DNA repair mechanisms in certain cancers. In terms of TMB, LPL is positively correlated in 5 tumor types including GBM, LAML, THYM, OV, and ACC, while it is negatively correlated in 13 cancer types including GBMLGG, LGG, LUAD, COAD, and COADREAD. This bidirectional correlation implies that LPL may have heterogeneous molecular functions in different tumor types or play different regulatory roles under different genetic mutation backgrounds. Notably, LPL exhibited strong positive correlations with mast cells, dendritic cells, and macrophages across LUAD and other cancer types while showing negative correlations with immunosuppressive cells such as NK cells and Th2 cells. This suggests that LPL may influence tumor immune evasion and local immune regulation by modulating immune activities within the tumor microenvironment. Such immunological relevance highlights the multifaceted roles of LPL in tumor immunity regulation and underscores its potential value in immunotherapy, particularly in its interactions with key immune checkpoint molecules like PD-1 and CTLA4, which could significantly influence treatment response rates. Furthermore, we observed that LPL expression was significantly negatively correlated with TMB but positively correlated with PD-L1 expression, which is not entirely consistent with the conventional view that high TMB and high PD-L1 levels jointly indicate better responsiveness to immunotherapy. One possible explanation is that, as suggested by immune cell infiltration analysis and single-cell transcriptomic data, LPL is predominantly expressed in the non-tumor components of the tumor microenvironment, particularly macrophages. Its high expression may reflect an immunosuppressive microenvironment characterized by reprogrammed lipid metabolism. Previous studies have shown that lipid accumulation can induce PD-L1 upregulation and suppress T cell effector functions through metabolic remodeling [55,56,57], which may account for the observed positive correlation between LPL and PD-L1. In contrast, TMB primarily reflects the quantity of tumor-specific neoantigens derived from somatic mutations and is less related to metabolic state, thereby exhibiting a negative correlation with LPL. Furthermore, although LPL expression was negatively correlated with PD-1, our immune-related score analysis (IPS) indicated that tumors with high LPL expression exhibited greater potential responsiveness to anti-PD-1 therapy. This suggests that LPL may influence immunotherapeutic sensitivity by modulating T cell metabolic states. Collectively, these findings indicate that LPL may serve as a critical link between lipid metabolism and immunosuppression and represent a potential metabolic–immune regulatory target worthy of further investigation. In the survival prognosis assessment, the Kaplan–Meier method indicated that a reduced LPL expression was correlated with poorer overall survival in LUAD cases and was significantly related to adverse clinical outcomes. LPL demonstrates significant diagnostic value in LUAD, as its expression differs notably from traditional immune markers such as TTF1, CK7, and NAPSA. However, the weak correlation between LPL and these markers suggests that LPL’s expression pattern is largely independent of the commonly used clinical immune markers. This highlights LPL’s unique role as a metabolic biomarker in LUAD. While TTF1, CK7, and NAPSA are routinely utilized for tumor origin identification and classification, LPL offers a novel perspective by reflecting changes in tumor metabolism and the tumor microenvironment. LPL’s expression is not directly associated with the differentiation status of tumor cells, but is more influenced by the metabolic activity of immune and stromal cells within the tumor microenvironment. Therefore, LPL’s independence from traditional markers positions it as a promising diagnostic tool, providing new insights for the precise diagnosis and treatment of LUAD. It should be noted that, although LPL exhibits strong diagnostic performance in LUAD and its expression is closely linked to the immune microenvironment, its AUC for independently predicting LUAD patient survival falls short of the desired threshold. This may be because patient survival in LUAD is governed by a multitude of factors—such as overall physiological status, adjuvant treatments, and tumor progression—of which LPL represents only one regulatory element. Similar phenomena have been observed for genes like DDB2 in breast cancer and IDH1 in glioblastoma. Consequently, we believe that the prognostic utility of LPL warrants further investigation using multivariate models and validation in well-characterized clinical cohorts. Changes in LPL expression were observed across different stages of lymph node metastasis, genders, and TP53 mutation groups, further supporting its potential as a prognostic biomarker. Through drug sensitivity analyses, we discovered that LPL expression levels were linked to the sensitivity of multiple small-molecule kinase inhibitors. Cell function verification experiments suggest that treating the LUAD A549, PC-9, and H1975 cell lines with the LPL agonist Ibrolipim significantly inhibits the proliferation and migration activities of the cells, as observed through the cell scratch assay, the CCK8 assay, and the IncuCyte live-cell imaging system. Meanwhile, in the investigation of the mechanism of LPL in LUAD cells, we demonstrated through qRT-PCR experiments that Ibrolipim dose-dependently upregulated LPL mRNA levels in A549 cells (the 100 μmol/L group was significantly upregulated compared to the control group, *p* < 0.01). This indicates that LPL expression and activity in lung tissue play an important role in the prevention and treatment of LUAD, and activating LPL expression may inhibit the metastasis or growth of LUAD. At the same time, we also observed the inhibitory effect of activating LPL on H1299 cells, suggesting that LPL may also play an important role in large-cell lung cancer. Furthermore, our comprehensive analysis indicates that the downregulation of tumor-specific LPL in LUAD may be attributed to transcriptional dysregulation. By integrating multiple transcription factor prediction databases and expression profile analyses from the TCGA and GTEx datasets, we found that the loss of JUND-mediated transcriptional activation might be the cause of the reduced LPL levels in tumors. These findings provide preliminary mechanistic insights into the regulation of LPL in LUAD. However, the specific mechanism underlying the downregulation of LPL in LUAD still requires further validation through biological experiments in subsequent studies. To further investigate the role of LPL in LUAD, we conducted comprehensive bioinformatics analyses to elucidate its multifaceted regulatory functions. Gene Set Enrichment Analysis (GSEA) revealed that low LPL expression is significantly enriched in pathways related to DNA synthesis, DNA replication, and cell cycle checkpoints, suggesting that LPL downregulation may promote tumor progression by facilitating cell cycle dysregulation and genomic instability. Additionally, Reactome pathway enrichment analysis of LPL-associated differentially expressed genes indicated significant involvement in lipid metabolism remodeling (such as chylomicron and plasma lipoprotein remodeling), epithelial differentiation (formation of the cornified envelope), and membrane potential signaling (Phase 0 rapid depolarization). These findings imply that LPL plays a crucial role in maintaining pulmonary epithelial metabolic homeostasis and barrier function. Collectively, our results suggest that LPL may contribute to LUAD pathogenesis through coordinated regulation of cell cycle control, lipid metabolism, and epithelial function remodeling, providing a novel mechanistic basis for its potential as a therapeutic target. Based on predictions from the drug database, it is known that for patients with tumors exhibiting low LPL expression, drugs such as trametinib and afatinib might exhibit enhanced therapeutic efficacy. This provides new insights into personalized treatment plans for LUAD and lays an experimental foundation for the future development of drugs targeting LPL in the treatment of LUAD.

In conclusion, this investigation explored the potential mechanisms underlying the role of the LPL gene in LUAD through comprehensive and diverse approaches. Bioinformatics studies and cell function experiments suggest the multifaceted roles of LPL in LUAD, including tumor occurrence, immune regulation, metabolic regulation, and targeted drug therapy. These findings provide new insights for future precision medical strategies based on the LPL target and lay a theoretical and experimental foundation for the development of new therapeutic targets. Although there have been many studies using IF or IHC techniques to detect the expression of LPL in various diseases such as gastric cancer [37] and NSCLC [36], there are no reports on the expression differences of LPL in normal tissues and LUAD tissues using IF or IHC techniques. This is also the focus of this study. However, due to the limitations of ethical approval procedures and sample acquisition conditions, we are currently unable to analyze the results of immunohistochemistry or immunofluorescence experiments on human LUAD tissue samples. In future studies, we will prioritize obtaining clinical LUAD tissue samples and combine IHC and multiplex immunofluorescence techniques to further verify the spatial co-localization relationship between LPL and immune cells and its regulatory role on the immune microenvironment, thereby strengthening the biological basis and clinical translational potential of the conclusions of this study. In subsequent studies, we aim to delve deeper into the precise molecular pathways of LPL involved in the development of LUAD, as well as its potential implementation in clinical therapy. This may offer fresh insights for the advancement of personalized and precision medicine.

## 5. Conclusions

In conclusion, our study has shown that reduced LPL expression in lung tissue may be linked to a higher risk of LUAD development. LPL is essential for the diagnosis of LUAD and is associated with the infiltration of distinct immune cell populations within LUAD tumors. LUAD patients with low LPL expression levels respond well to immune checkpoint therapy, and activating LPL in cell experiments can inhibit the activity of LUAD cells. Therefore, we suggest that LPL may serve as a novel biomarker for LUAD, and targeting LPL may have certain value in the treatment of LUAD.

## Data Availability

The dataset used and analyzed in this study is available from the corresponding author upon reasonable request.

## Supplementary Materials

The following supporting information can be downloaded at: https://www.mdpi.com/article/10.3390/biology14050566/s1, Figure S1. The correlation between LPL and the expression of common clinical immune markers. (a) The correlation between LPL and TTF1 expression in LUAD. (b) The correlation between LPL and NAPSA expression in LUAD. (c) The correlation between LPL and CK7 expression in LUAD. Figure S2. The activator of LPL, apoC II, can inhibit the proliferation activity and migration ability of the lung adenocarcinoma A549 cell line. (a) Cell viability of A549 cells treated with different doses of apoC II for 24 h. (b) Comparison of the migration rate of A549 cells in different apoC II treatment groups. (c) Migration of A549 cells treated with different doses of apoC II for 24 h. Figure S3. The LPL activator, Ibrolipim, has the ability to suppress both the proliferative capacity and migratory potential of LUAD cells. (a) The viability of H1299 cells was assessed after treatment with varying concentrations of Ibrolipim for 24 h. (* indicates significant differences between each dose group and the Control group. * *p* < 0.05, ** *p* < 0.01, *** *p* < 0.001) (b) Comparison of migration rates of H1299 cells in different treatment groups.(# indicates significant differences between each dose group and the Control group at 12 h, * indicates significant differences between each dose group and the Control group at 24 h. * *p* < 0.05, ** *p* < 0.01, *** *p* < 0.001; # *p* < 0.05, ## *p* < 0.01, ### *p* < 0.001) (c) The relative change curve of cell area in each group of the H1299 cell line. (e) The observation results of live cells in each group of the H1299 cell line at 0, 12, and 24 h. (* *p* < 0.05, ** *p* < 0.01, *** *p* < 0.001; # *p* < 0.05, ## *p* < 0.01, ### *p* < 0.001). Figure S4. The relative expression levels of LPL mRNA in different dose Ibrolipim treatment groups. Figure S5. The 20 most significant pathways in GSEA analysis. All *p* values are 1^−10^. Figure S6. The expression analysis results of LPL in single-cell samples. (a) The relative expression level of LPL in GSE117570; (b) The clustering of single-cell results in GSE117570; (c) The violin plot of LPL expression in relevant cells in GSE117570. (d) The relative expression level of LPL in GSE127465; (e) The clustering of single-cell results in GSE127465; (f) The violin plot of LPL expression in relevant cells in GSE127465. (g) The relative expression level of LPL in E-MTAB-6149; (h) The clustering of single-cell results in E-MTAB-6149; (i) The violin plot of LPL expression in relevant cells in E-MTAB-6149.

## Abbreviations

ALK	Anaplastic Lymphoma Kinase
AUC	Area Under Curve
CancerSEA	Cancer Single-Cell State Atlas
CCK-8	Cell Counting Kit-8
CPADS	Cancer Personalized Single-cell Atlas Data Server
CTRP	Cancer Therapeutics Response Portal
DEGs	Differentially Expressed Genes
DGIdb	Drug-Gene Interactions and the druggable genome
EGFR	Epidermal Growth Factor Receptor
eQTLs	Expression Quantitative Trait Loci
FBS	Fetal Bovine Serum
FDR	False Discovery Rate
GEO	Gene Expression Omnibus
GTEx	Genotype-Tissue Expression project
GWAS	Genome-Wide Association Study
HEIDI	Heterogeneity in Dependent Instruments
ICI	Immune Checkpoint Inhibitor
ILCCO	International Lung Cancer Consortium
IPS	Immunophenoscore
LPL	Lipoprotein Lipase
LUAD	Lung Adenocarcinoma
MSI	Microsatellite Instability
NCCN	National Comprehensive Cancer Network
NSCLC	Non-Small-Cell Lung Cancer
OR	Odds Ratio
PPH4	Posterior Probability of Hypothesis 4
ROC	Receiver Operating Characteristic
scRNA-seq	Single-cell RNA sequencing
SMR	Summary-data-based Mendelian Randomization
SNPs	Single Nucleotide Polymorphisms
ssGSEA	single-sample Gene Set Enrichment Analysis
TCGA	The Cancer Genome Atlas
TCIA	The Cancer Immunome Atlas
TIMER	Tumor Immune Estimation Resource
TMB	Tumor Mutational Burden
TRICL	Transdisciplinary Research in Cancer of the Lung
UALCAN	University of Alabama Cancer Database
