# Supplementary material for: Integrated Bioinformatics Analysis and Cellular Experimental Validation Identify Lipoprotein Lipase Gene as a Novel Biomarker for Tumorigenesis and Prognosis in Lung Adenocarcinoma
Source: Biology (Basel). 2025 May 19;14(5):566. doi: 10.3390/biology14050566 (PMC12108960; doi:10.3390/biology14050566)
Supplement: Supplementary file 1 [file biology-14-00566-s001.zip › biology-3595975-supplementary.pdf]

Supplementary Materials

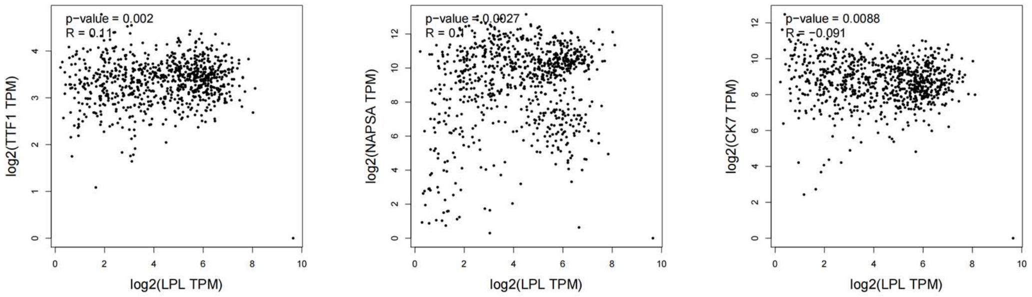

(**a**) (**b**) (**c**)

Figure S1. The correlation between LPL and the expression of common clinical immune markers. (a) The correlation between LPL and TTF1 expression in LUAD. (b) The correlation between LPL and NAPSA expression in LUAD. (c) The correlation between LPL and CK7 expression in LUAD.

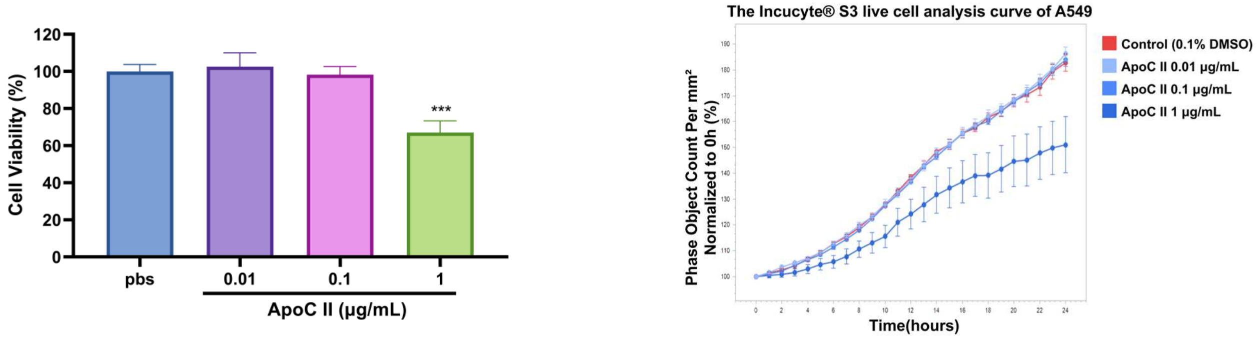

(**a**) (**b**)

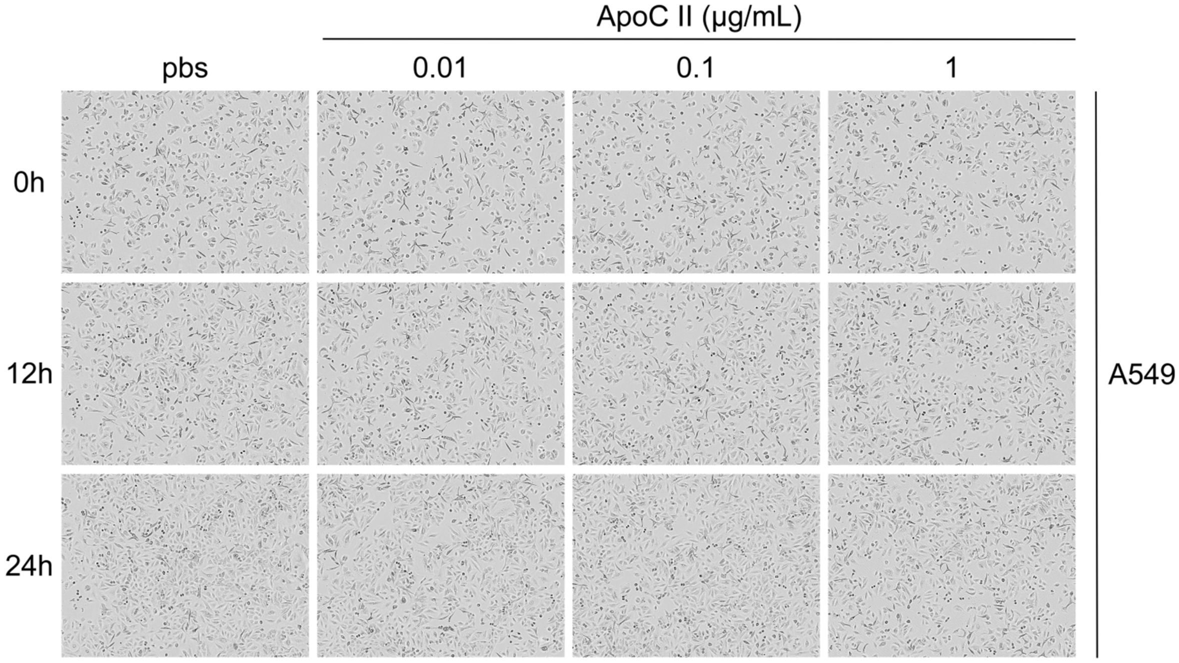

(**c**)

Figure S2. The activator of LPL, apoC II, can inhibit the proliferation activity and migration ability of the lung adenocarcinoma A549 cell line. (a) Cell viability of A549 cells treated with different doses of apoC II for 24 hours. (b) Comparison of the migration rate of A549 cells in different apoC II treatment groups. (c) Migration of A549 cells treated with different doses of apoC II for 24 hours.

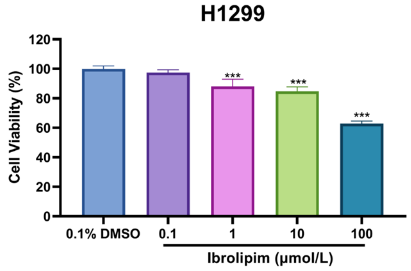

(a)

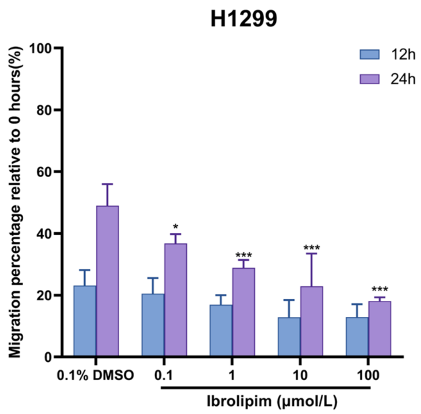

(b)

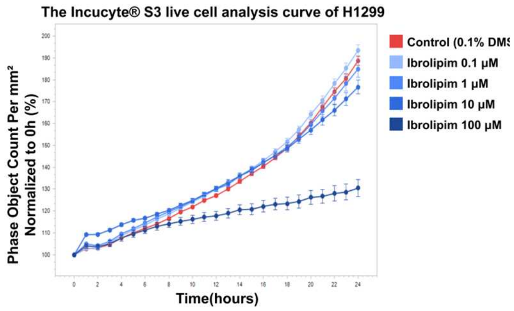

(c)

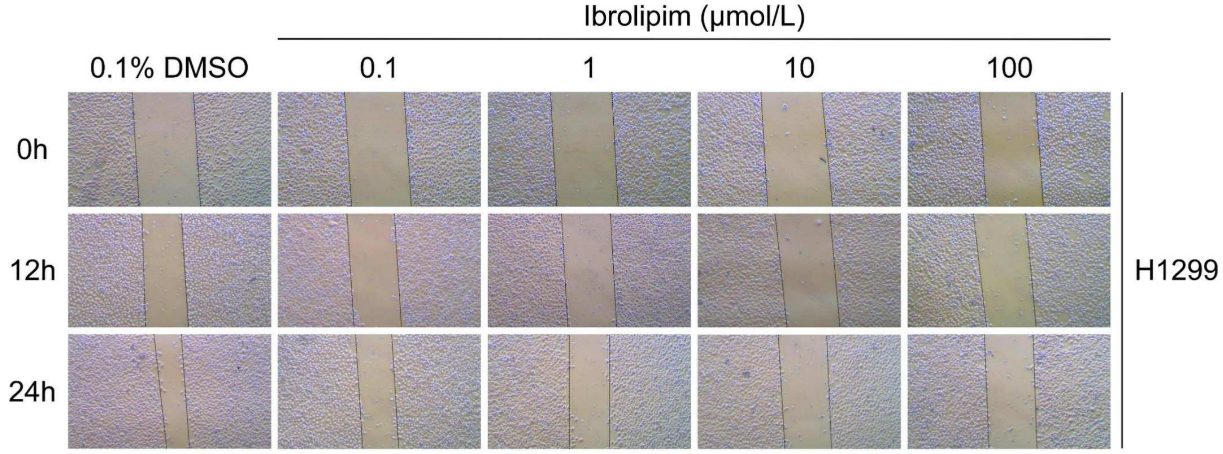

(d)

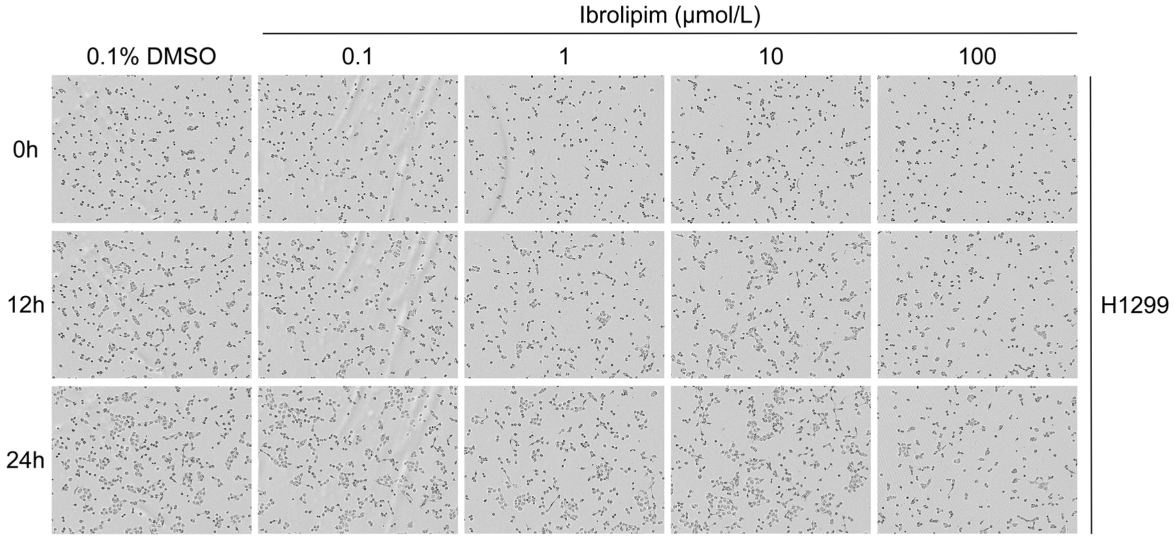

(e)

Figure S3. The LPL activator, Ibrolipim, has the ability to suppress both the proliferative capacity and migratory potential of LUAD cells. (a) The viability of H1299 cells was assessed after treatment with varying concentrations of Ibrolipim for 24 hours.(* indicates significant differences between each dose group and the Control group. * $P < 0.05$, ** $P < 0.01$, *** $P < 0.001$) (b) Comparison of migration rates of H1299 cells in different treatment groups.(# indicates significant differences between each dose group and the Control group at 12h, * indicates significant differences between each dose group and the Control group at 24h. * $P < 0.05$, ** $P < 0.01$, *** $P < 0.001$; # $P < 0.05$, ## $P < 0.01$, ### $P < 0.001$) (c) The relative change curve of cell area in each group of the H1299 cell line. (e) The observation results of live cells in each group of the H1299 cell line at 0, 12, and 24 hours. (* $P < 0.05$, ** $P < 0.01$, *** $P < 0.001$; # $P < 0.05$, ## $P < 0.01$, ### $P < 0.001$)

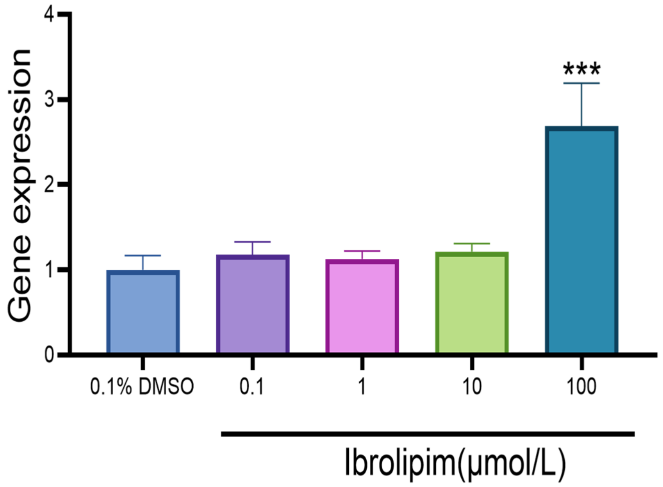

Figure S4. The relative expression levels of LPL mRNA in different dose Ibrolipim treatment groups

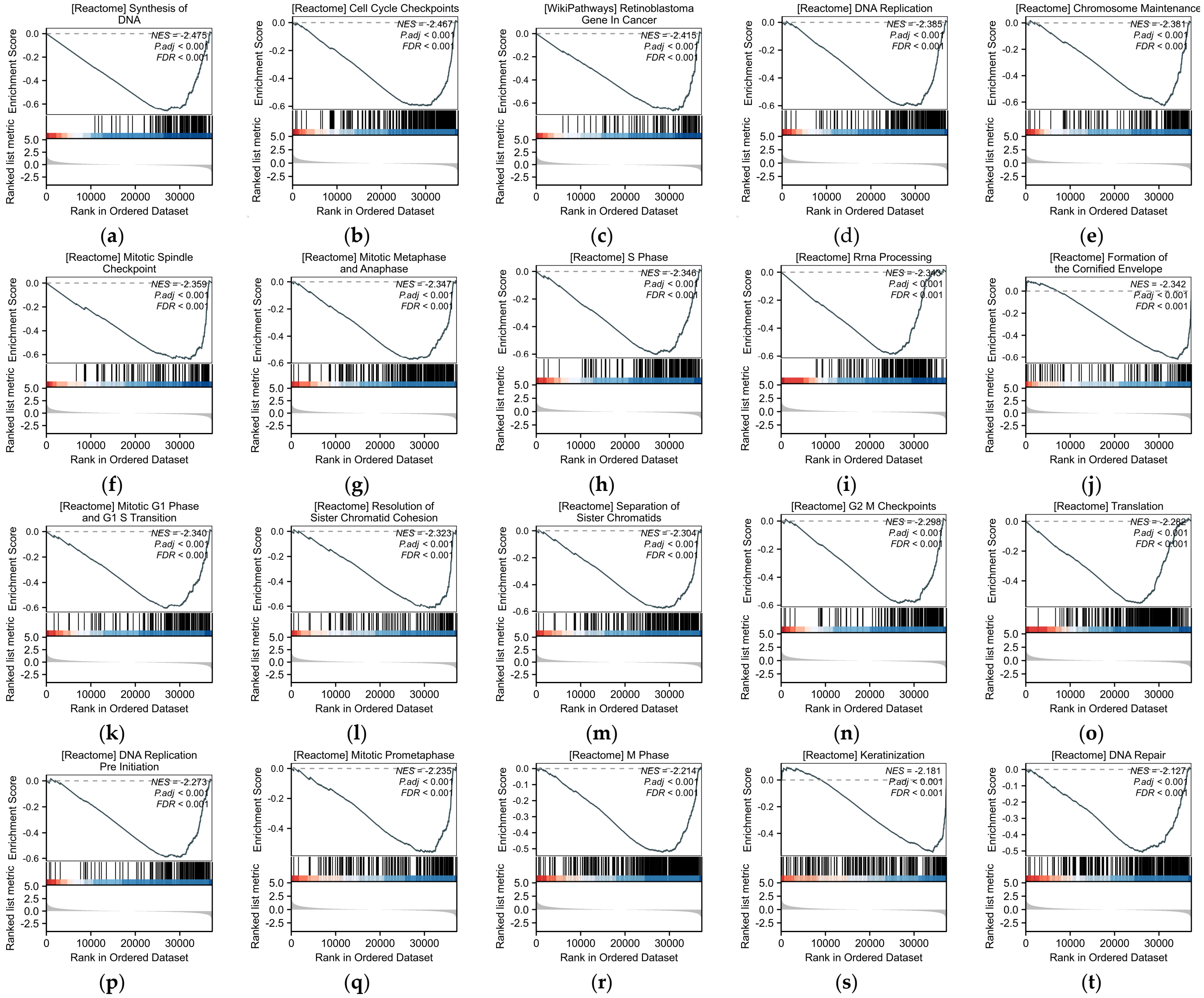

Figure S5. The 20 most significant pathways in GSEA analysis. All P values are 1^{-10}.

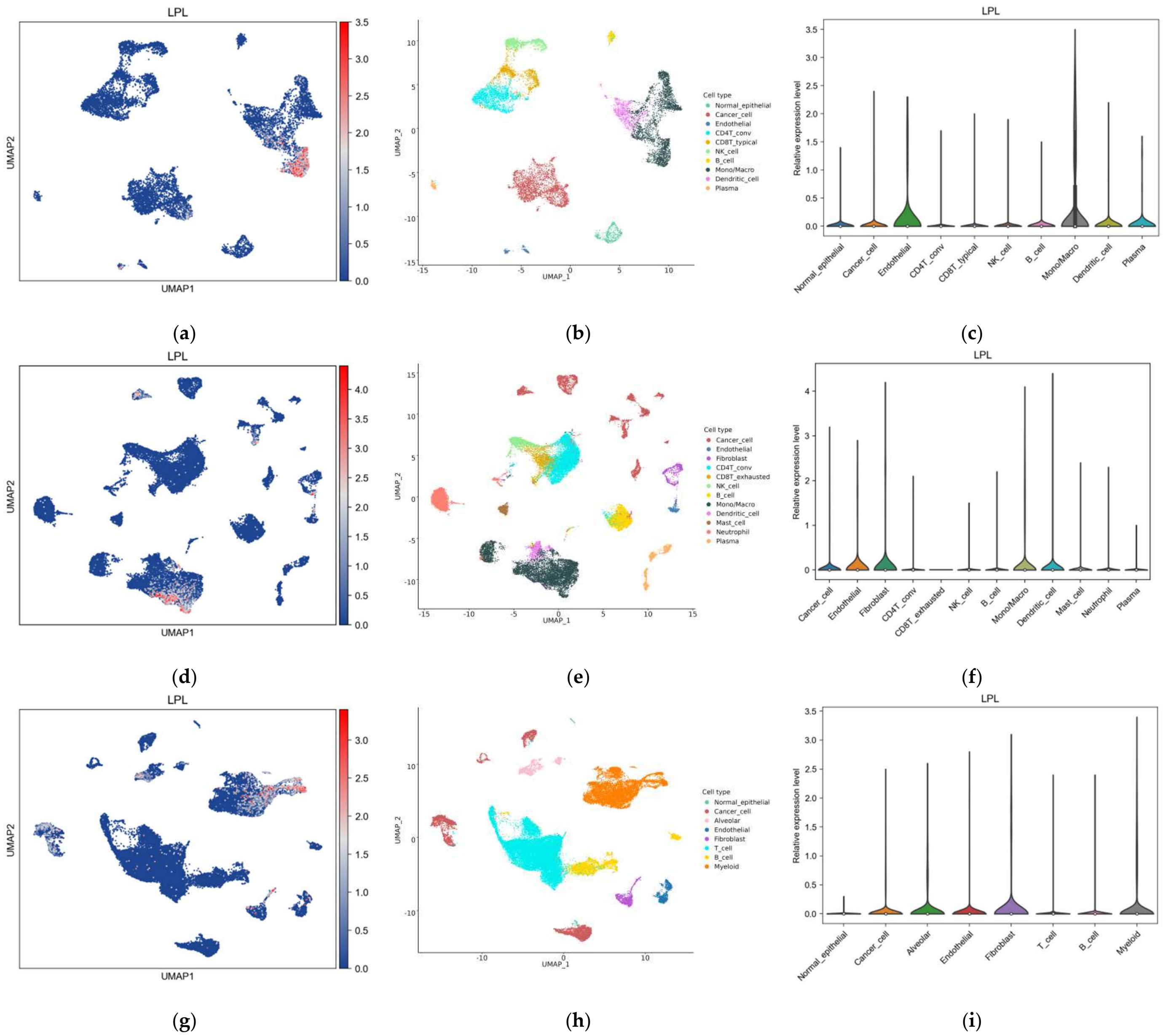

Figure S6. The expression analysis results of LPL in single-cell samples. (a) The relative expression level of LPL in GSE117570; (b) The clustering of single-cell results in GSE117570; (c) The violin plot of LPL expression in relevant cells in GSE117570. (d) The relative expression level of LPL in GSE127465; (e) The clustering of single-cell results in GSE127465; (f) The violin plot of LPL expression in relevant cells in GSE127465. (g) The relative expression level of LPL in E-MTAB-6149; (h) The clustering of single-cell results in E-MTAB-6149; (i) The violin plot of LPL expression in relevant cells in E-MTAB-6149.
